# Tead transcription factors differentially regulate cortical development

**DOI:** 10.1038/s41598-020-61490-5

**Published:** 2020-03-13

**Authors:** Tanzila Mukhtar, Jeremie Breda, Alice Grison, Zahra Karimaddini, Pascal Grobecker, Dagmar Iber, Christian Beisel, Erik van Nimwegen, Verdon Taylor

**Affiliations:** 1 Department of Biomedicine, University of Basel, Mattenstrasse 28, CH-4058 Basel, Switzerland; 2 Biozentrum, University of Basel, Klingelbergstrasse 50-70, CH-4056 Basel, Switzerland; 3 Swiss Institute of Bioinformatics (SIB), Mattenstrasse 26, CH-4058 Basel, Switzerland; 4 Computational Biology Group, Department of Biosystems Science and Engineering, ETH Zürich, Mattenstrasse 26, CH-4058 Basel, Switzerland; 5 Department of Biosystems Science and Engineering, Mattenstrasse 26, ETH Zürich, CH-4058 Basel, Switzerland

**Keywords:** Neural stem cells, Developmental neurogenesis

## Abstract

Neural stem cells (NSCs) generate neurons of the cerebral cortex with distinct morphologies and functions. How specific neuron production, differentiation and migration are orchestrated is unclear. Hippo signaling regulates gene expression through Tead transcription factors (TFs). We show that Hippo transcriptional coactivators Yap1/Taz and the Teads have distinct functions during cortical development. Yap1/Taz promote NSC maintenance and Satb2^+^ neuron production at the expense of Tbr1^+^ neuron generation. However, Teads have moderate effects on NSC maintenance and do not affect Satb2^+^ neuron differentiation. Conversely, whereas Tead2 blocks Tbr1^+^ neuron formation, Tead1 and Tead3 promote this early fate. In addition, we found that Hippo effectors regulate neuronal migration to the cortical plate (CP) in a reciprocal fashion, that *ApoE*, *Dab2* and *Cyr61* are Tead targets, and these contribute to neuronal fate determination and migration. Our results indicate that multifaceted Hippo signaling is pivotal in different aspects of cortical development.

## Introduction

NSCs of the developing cerebral cortex form the ventricular zone (VZ) lining the lumen of the neural tube^1–5^. NSCs in the dorsal anterior forebrain are the major source of the projection neurons of the cerebral cortex^4,5^. The mechanisms controlling the patterning and cell fate specification of these stem cells during early brain development are not clearly understood. Although various signaling pathways including Notch, Wnt, Shh, FGFs, TGF-β, Retinoic acid, Reelin and Hippo are known to regulate NSC proliferation and to control fate decisions, neurogenesis, and gliogenesis; the crosstalk between the different signaling pathways and the integration of these signals on target genes governing complex cell fate choices is unclear^1–3^.

Hippo signaling is evolutionarily conserved and a regulator of organ size control and tissue homeostasis^6–9^. The pathway is regulated by numerous stimuli including G-protein coupled receptor signaling, mechanical stress, cellular energy status, cell-cell contact and cell-extra-cellular matrix interactions^6–8^. Hippo signaling employs a cascade of phosphorylation steps mediated by the kinases Mst1/2 and Lats1/2^8–10^. Lats1/2 phosphorylate the transcriptional coregulators Yap1 and Taz to promote cytoplasmic retention and subsequent degradation^6–8^. When Hippo signaling is inactive, Yap1/Taz translocate to the nucleus and form multiple complexes with different DNA binding partners including TEADs, SMADs, and Runx TFs (Fig. S1a)^8–10^. The Teads are major regulators of Hippo target genes in many systems including cancer^8,11,12^.

Fat4 and Dchs are receptor and ligand, respectively, of the Hippo pathway in embryonic NSCs. Knockdown of Fat4 results in increased proliferation in the developing nervous system and reduction of neuronal differentiation^13,14^. Mutations in *FAT4* and DCHS cause Van Maldergem syndrome in humans, an autosomal-recessive disorder characterized by intellectual disability, auditory, craniofacial, skeletal, limb and renal malformations^14^. In many cases, Van Maldergem syndrome is associated with reduced cortical volume and a partially penetrant formation of periventricular neuronal heterotopias caused by miss-localized neurons in the periventricular area of the forebrain^13,15^. Therefore, Hippo signaling potentially plays a role in gyrification in higher vertebrates^15^. Manipulation of *Fat4* and *Dchs* expression in the developing mouse cerebral cortex replicated some aspects of Van Maldergem syndrome^14^. However, the downstream molecular mechanisms are still not known, particularly in the light that Yap1 localization was not obviously affected in *Fat4, Dchs* double-mutant mice and Fat4 may not be able to activate Hippo signaling in some cell-types^14,16^.

*Yap1*^−/−^ mice developmentally arrest during mid-embryogenesis and die, precluding analysis of Yap1 function in brain development^17,18^. Conditional gene ablation from the progenitors of the developing nervous system shows Yap1 to be necessary for ependymal progenitor cell formation and the mice develop hydrocephaly soon after birth^17,18^. Conversely, *Taz*^−/−^ mice are viable but develop renal cysts and lung defects^19^. Comparatively little is known about Taz functions in the developing brain. Overexpression experiments expressing Yap1 and Taz in NSCs implied that Tead2 is the mediator in their control of neural progenitor proliferation and neurogenesis^20^. *Tead1*^−/−^ and *Tead2*^−/−^ mice show severe growth retardation and morphological abnormalities including failure in dorsal neural tube closure as well as notochord and somite defects^21,22^. However, analysis also revealed partial redundancy in Tead1/2 functions during early development^21,22^. Although *Fat1*/*Fat4* double knockout mice show similar neural tube closure defects suggesting redundancy in these two receptors, the downstream mechanisms causing these phenotypes are not understood^13^.

In this study, we addressed the functions of the Hippo effectors, the Teads, during mouse cortical development. We find that the expression of Hippo signaling components is highly dynamic during cortical development within the NSC, basal progenitor (BP) and neuronal lineages. Whereas in many systems Tead factors are redundant^21^, they show specific cell-type and temporal dynamics in their expression during cortical development. We show by gain and loss of function experiments that Tead1 and Tead3 are functionally similar but their effects on cortical development are distinct to that of Tead2. Using Integrated Motif Activity Response Analysis (ISMARA), we predicted Tead targets and validated direct targets in NSCs by ChIP and expression analyses *in vivo*^23^. We show that ApoE, Cyr61 and Dab2, which regulate activity of the Reelin receptor ApoER2, partially convey the Tead-mediated mutant phenotypes we observed during cortical development. Thus, our data indicate multiple and specific roles of Hippo signaling effectors during cortical neurogenesis and provide a link between the Hippo and the Reelin pathways.

## Results

### Hippo signaling effectors are dynamically expressed during cortical development

**Figure 1:**
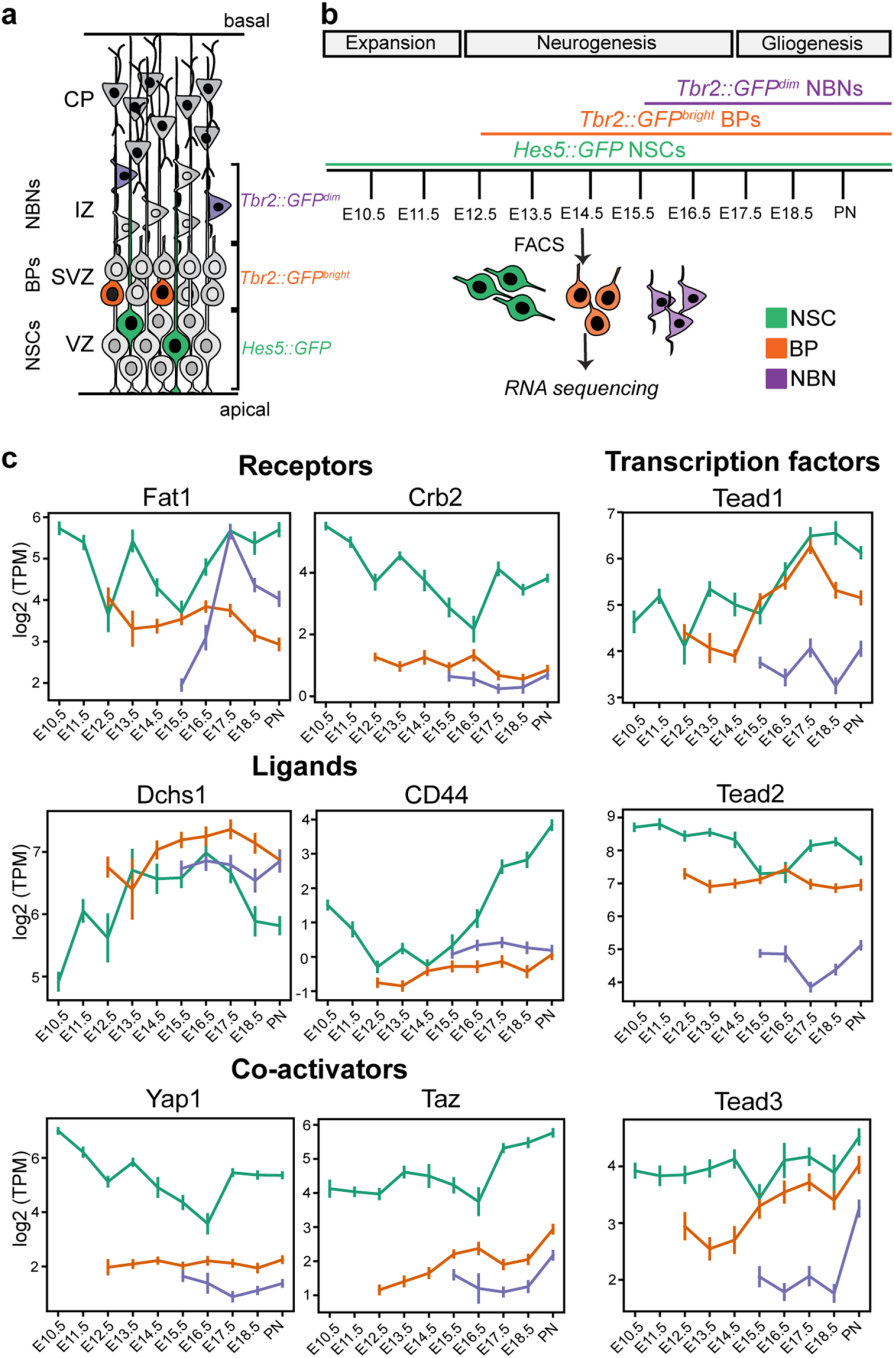
Transcriptional dynamics of Hippo effectors in NSCs, BPs, NBNs from RNA sequencing data. (**a**) Schematic representation of mouse developing cortex. NSCs reside in the VZ, with long processes extending from apical to basal surface. NSCs are labelled by *Hes5::GFP*. BPs express high levels of *Tbr2::GFP* (*Tbr2::GFP*^bright^) and NBNs express low levels of *Tbr2::GFP* (*Tbr2::GFP*^dim^) expression. (**b**) Experimental paradigm used. 3–4 RNA samples extracted from FAC-sorted GFP^+^ NSCs, BPs and NBN populations each day during development, from biological replicates, following the time-course (E10.5 to PN), through phases of expansion, neurogenesis and gliogenesis. cDNA libraries were prepared and Next-Generation RNA-sequencing performed. (**c**) Expression profiles of Hippo signaling effectors; receptors Fat1 and Crb2, ligands Dchs1, CD44, co-activators Yap1, Taz, transcription factors Tead1, Tead2, Tead3 in NSCs, BPs and NBNs show dynamics during corticogenesis in these populations. Y-axis: mRNA level expressed as log2 TPM (transcripts per million). Also see Figs. S1 and S2. NSCs- Neural stem cells, BPs- Basal progenitors, NBNs- Newborn neurons, VZ - ventricular zone, SVZ - subventricular zone, IZ - intermediate zone, CP - cortical plate, E - Embryonic day, PN - postnatal day 1.

To address the changes in gene expression by NSCs, BPs and post-mitotic newborn neurons (NBNs) during formation of the mouse dorsal cerebral cortex, we used the transgenic mouse lines *Hes5::GFP* and *Tbr2::GFP* to isolate pure populations of NSCs, BPs and NBNs between embryonic day 10.5 (E10.5) and birth (PN) (Figs. 1a and S1b–d) (Mukhtar *et al*. manuscript in preparation)^24,25^. Acute staining of the sorted cells validated the purity of the selected NSC, BP and NBN populations (Fig. S1e–g). E10.5-PN covered the embryonic stages of cortex development from NSC expansion (E10.5-E11.5), through neurogenesis (E12.5–E16.5) to gliogenesis (E17.5-PN) (Fig. 1b). Transcriptome analysis revealed dynamic expression of Hippo signaling components during corticogenesis (Figs. 1c and S2a,b, Supplementary Data File – RNA-Seq tab). The downstream effectors of Hippo signaling, the Teads, showed distinct and dynamic expression at the mRNA level indicative of potential specific functions. *Tead1* and *Tead2* expression were partially reciprocal in NSCs. While *Tead1* expression increased from the expansion and neurogenic to the gliogenic phase, *Tead2* was expressed highest by expanding NSCs and reduced during late neurogenesis (Fig. 1c). *Tead3* expression remained relatively constant in NSCs during all phases and *Tead4* mRNA was not detected at significant levels during cortical development (Figs. 1c and S2b). In BPs, the expression of the different *Tead* genes was also distinct and dynamic. *Tead1* and *Tead3* were expressed at lower levels by BPs at early stages (E12.5–E14.5) but increased dramatically at later stages (E15.5-PN). Conversely, *Tead2* mRNA was expressed at high levels by BPs of all stages (Fig. 1c). These findings suggested that Teads have distinct temporal and cell-type specific functions during cortical development.

The genes of the Tead co-activators Yap1 and Taz showed dynamic and partially reciprocal expression by dorsal cortical NSCs. *Yap1* expression paralleled *Tead2*, reducing during late neurogenesis while *Taz* expression was more similar to that of *Tead1*, increasing during the gliogenic phase of cortical development and in late stage BPs (Fig. 1c). Immunocytochemical analysis confirmed that the Yap1 and Tead1 proteins were expressed across cortical development, predominantly by the *Sox2*^+^ progenitors in the VZ (Fig. S2c). Thus, expression analysis suggested that Yap1 and Taz could use different Teads to transduce Hippo signals to target genes. Hippo signaling is activated by a variety of upstream receptors including Fat and Crb family members. NSCs expressed *Fat1* and *Crb2* with similar dynamics with lower expression during neurogenesis, while *Fat3* and *Fat4* expression were higher during the neurogenic phase compared to the expansion and gliogenic phases of corticogenesis (Figs. 1c and S2b). Hippo receptors also showed distinct dynamic expression in BPs and NBNs (Figs. 1c and S2b). *Fat1* was expressed highly by BPs and NBNs while *Crb2* was predominantly expressed by NSCs (Figs. 1c and S2b). The genes of the Hippo ligands Dchs1 and CD44 also showed different dynamics in expression. *CD44* was expressed by NSCs but not BPs or NBNs. Conversely, *Dchs1* was expressed at high levels by all cell-types of the lineage (Fig. 1c). This indicated that Hippo signaling in the progenitors of the developing cortex is complex and could be dynamic over time and through the lineage with different receptors, ligands and downstream components being utilized to communicate between different cell-types.

### Yap1/Taz overexpression in NSCs affects cortical layering

**Figure 2:**
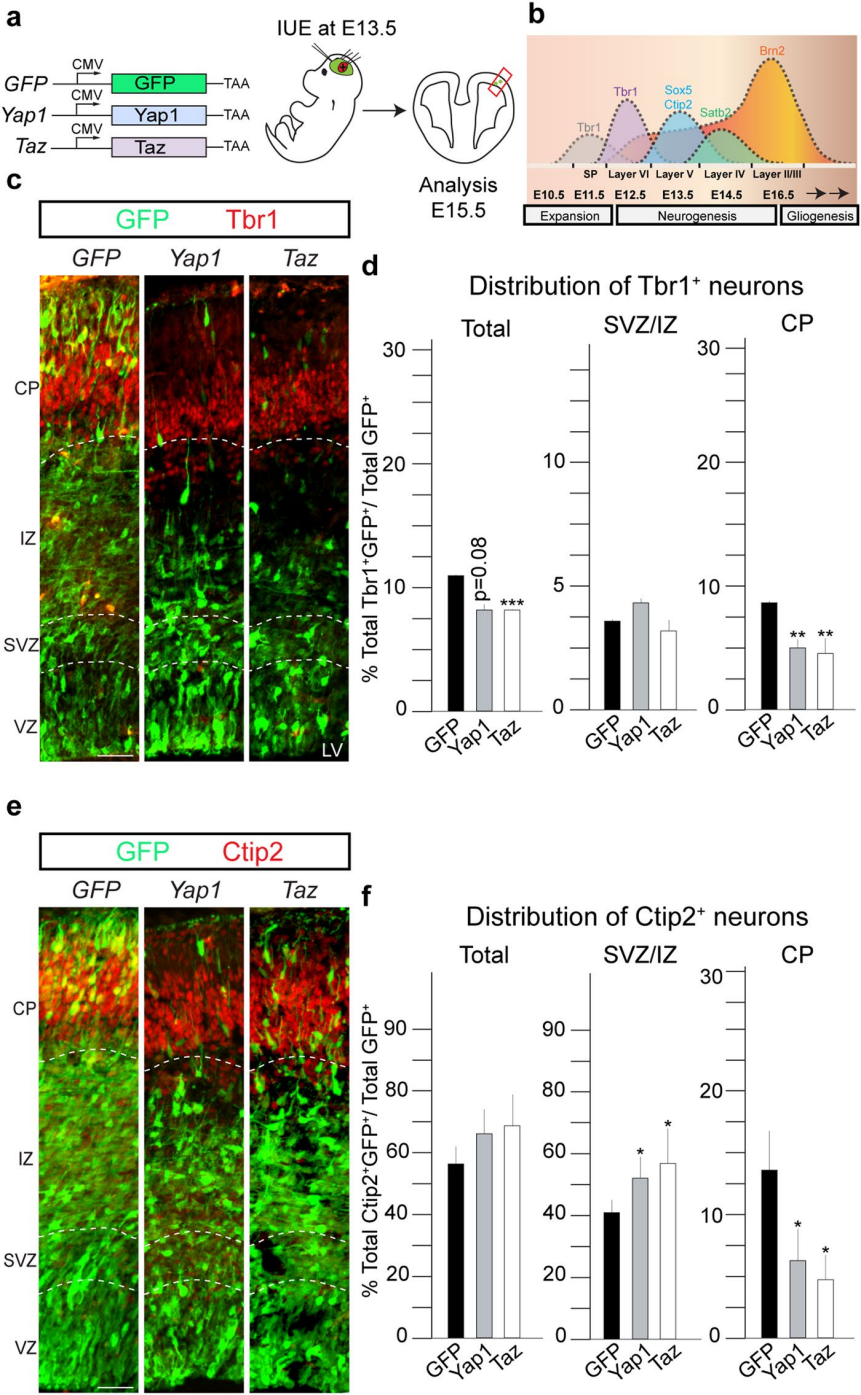
Overexpression of co-activators Yap1 and Taz affects cell fate, neuronal migration and cortical layering. (**a**) Experimental paradigm used to perform overexpression of Yap1 and Taz, and GFP as a control. IUEs were performed at E13.5 and brains isolated at and analyzed at E15.5, after 48 hours. (**b**) Illustration to show the sequential generation of distinct types of cortical layers, specified by different TFs. The cortical development is divided in expansion, neurogenesis and gliogenesis. (**c**) Coronal sections of transfected cortices immunostained for GFP and Tbr1, deep layer marker. (**d**) Quantification of Tbr1^+^GFP^+^ cells shows a reduction upon overexpression of Yap1 and Taz, compared to GFP control in CP and total. (**e**) Coronal sections of transfected cortices immunostained for GFP and Ctip2, deep layer marker. (**f**) Quantification of Ctip2^+^GFP^+^ cells shows a reduction compared to GFP control in CP and no change in total, upon overexpression of Yap1 and Taz. IUE- *in utero* electroporation. Total = VZ+SVZ/IZ+CP. Also see Fig. S3. Summaries of the quantifications are in Table S1. Scale bar = 50 μm. Data are shown as average ± SEM, *p = 0.05, **p = 0.01, ***p = 0.001.

In order to address the function of Hippo signaling in the generation of cortical neurons during development, we used *in utero* electroporation (IUE) to force expression of Yap1 and Taz in NSCs *in vivo* (Fig. 2a,b). Expression of Yap1 or Taz resulted in a cell-autonomous retention of cells (GFP^+^) in the VZ 48 hours after electroporation compared to GFP expressing control cells (Fig. S3a–c). This cellular retention in the VZ was associated with an increase in Pax6^+^GFP^+^ cells in the VZ and in the subventricular zone (SVZ) (Fig. S3c). In parallel, there was a reduction in Yap1 and Taz overexpressing cells migrating to the CP (Fig. S3b). We asked whether the increase in Pax6^+^GFP^+^ induced by overexpression of Yap1 and Taz was due to the increase in cell proliferation. However, Ki67^+^GFP^+^ or pHH3^+^GFP^+^ cells were not significantly changed in the apical VZ following Yap1 or Taz overexpression (Fig. S3d–g). Yap1 overexpression did significantly increase Ki67^+^GFP^+^ cells in the SVZ/intermediate zone (IZ) compared to the control (Fig. S3d–g). We analyzed whether expression of Yap1 or Taz affected cell survival. Taz overexpression resulted in an increase in cleaved-Caspase-3^+^ cells in SVZ/IZ, but cell death was not increased upon Yap1 overexpression (Fig. S3h,i and data not shown).

We addressed whether the increase in Pax6^+^ cells upon overexpression of Yap1 and Taz resulted in changes in Tbr2^+^ BP numbers. Taz but not Yap1 overexpression reduced Tbr2^+^GFP^+^ cells in the VZ (Fig. S3j,k). Tbr2^+^GFP^+^ cells in the SVZ/IZ were unaffected by either Yap1 or Taz manipulation. We also addressed whether the reduction in Yap1 and Taz expressing cells in the CP was associated with changes in the expression of neuronal markers. Overexpression of Yap1 and Taz reduced the total number of deep-layer Tbr1^+^ neurons generated and their seeding in the CP (12.3 ± 0.1% in the GFP control compared to 8.1 ± 1.7% in Yap1 and 8.3 ± 0.3% in Taz overexpressing animals; Figs. 2c,d and S3l). Similarly, Yap1 and Taz overexpression reduced Ctip2^+^ and Satb2^+^ neurons in the CP (Figs. 2e,f and S3l–n). However, the total proportion of transfected cells that expressed Ctip2 was not changed following Yap1 or Taz overexpression, indicating a putative migration defect of neurons from the SVZ/IZ to the CP (Figs. 2e,f and S3l). Interestingly, expression of either Yap1 or Taz increased the proportion of transfected cells that expressed Satb2. However, their migration to the CP was also diminished and they accumulated in the SVZ/IZ (Fig. S3m). Together, these data suggest that Yap1 and Taz maintain NSC character and regulate differentiation and migration (Table S1). The reduction in deep layer neurons (Tbr1^+^) suggests that Yap1 and Taz can alter NSC fate choices. Our findings are supported by previous reports showing disruption of the migration of NSC progeny in the developing cortex upon overexpression of Yap1 and Taz^21,26,27^.

### Tead1, Tead2 and Tead3 induce different effects on NSCs and cell migration

**Figure 3:**
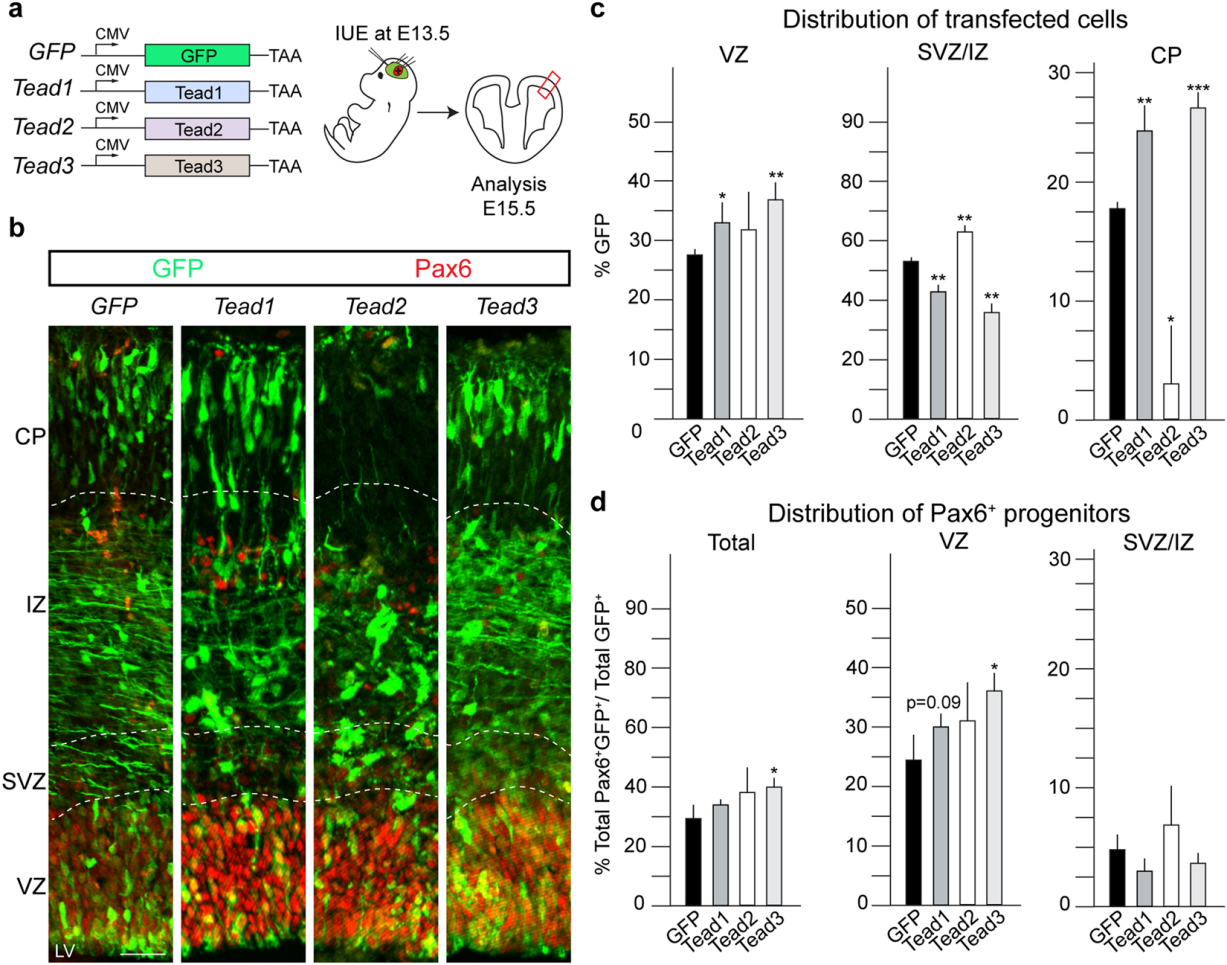
Overexpression of Tead1, Tead2, Tead3 affect cell fate, neuronal migration and cortical layering. (**a**) Experimental paradigm used to perform overexpression of Tead1, Tead2 and Tead3 and empty GFP as a control. IUE were performed at E13.5 and brains isolated at E15.5, after 48 hours. (**b**) Coronal sections of transfected cortices immunostained for GFP and Pax6, NSC marker. (**c**) Quantification of distribution of GFP^+^ transfected cells shows Tead1 and Tead3 induce similar phenotypic changes in cell distribution while Tead2 overexpression shows an opposite phenotype. (**d**) Quantification of Pax6^+^GFP^+^ cells shows an increase in total Pax6^+^ cells upon overexpression of Tead3, compared to GFP control in VZ and total. Also see Fig. S4. Summaries of the quantifications are in Table S2. Scale bar = 50 μm. Data are shown as average ± SEM, *p = 0.05, **p = 0.01, ***p = 0.001.

As Yap1 and Taz overexpression resulted in similar phenotypes, we addressed whether the Tead TFs are functionally comparable. We performed overexpression of Tead1, Tead2 and Tead3 (Fig. 3a). 48 hours after IUE, Tead1 and Tead3 expression significantly increased retention of transfected cells in the VZ and reduced cells in the SVZ/IZ (Fig. 3b,c). Tead3 increased the proportion of Pax6^+^GFP^+^ and also the proportion of GFP^+^ cells specifically in the VZ (Tead3; 41.2 ± 2.0%, over 30.6 ± 2.9% in GFP control; Fig. 3d). Tead1 and Tead3 overexpressing cells were, however, reduced in the SVZ/IZ and proportionally increased in the CP compared to controls suggesting premature migration from the SVZ/IZ to the CP (Tead1; 24.4 ± 1.3% and Tead3; 26.3 ± 0.8% compared to 17.9 ± 0.6% in control animals). In contrast, expression of Tead2 increased the number of cells in the SVZ/IZ and blocked their migration to the CP (Fig. 3b,c). The differential distribution of cells in the overexpression experiments indicated that Tead1, Tead2 and Tead3 can convey unique functions and potentially mediate independent downstream mechanisms in NSCs and their progeny.

We then addressed whether the Tead3-induced changes in Pax6^+^ cells were due to an increase in proliferation. Ki67^+^GFP^+^ cells were increase in the VZ following Tead3 overexpression but not after Tead1 or Tead2 expression (Fig. S4a,b). However, all of the Teads increased Ki67^+^GFP^+^ in SVZ/IZ (Fig. S4a,b). Moreover, only Tead3 increased pHH3^+^GFP^+^ apical NSCs lining the lumen of the telencephalic vesicle (Fig. S4c,d). We did not observe cell death (cleaved-Caspase-3^+^ cells) following overexpression of Tead1, Tead2 or Tead3 (data not shown).

### Tead1, Tead2 and Tead3 differentially affect neuronal fate

We analyzed changes in cell fate 48 hours after overexpression of the Tead TFs. Tead1, Tead2 and Tead3 overexpression did not affect the generation of Tbr2^+^ cells (Fig. S4e,f). Tead1 and Tead3 expression significantly increased differentiation of progenitors into Tbr1^+^ neurons (Fig. S4g,h). This increase was evident in both the SVZ/IZ and CP (Fig. S4g,h). However, Tead2 overexpression resulted in an almost complete block of Tbr1 neuron production (Fig. S4g,h). Therefore, we analyzed the expression of Ctip2, another marker of deep cortical layer neurons. Although the total proportion of transfected cells that expressed Ctip2^+^ was not changed, Tead1 overexpression increased Ctip2^+^ neurons in the CP (Fig. S4i,j). Whereas Tead3 also resulted in a trend towards an increase in CP Ctip2^+^ neurons, Tead2 overexpression dramatically reduced Ctip2^+^ neurons in the CP and slightly increased their appearance in the SVZ/IZ (Fig. S4i,j). In summary, Tead2 expression blocked Tbr1 but not Ctip2 fate acquisition and affected migration of both neuron populations to the CP.

Due to the effects of the Teads on deep layer neuron generation and migration, we analyzed expression of the upper layer neuronal marker Satb2 in response to Tead overexpression. None of the Teads induce changes in the proportion of cells that adopted a Satb2^+^ fate when overexpressed (Fig. S4k,l). However, Tead2 expression resulted in a dramatic reduction in Satb2^+^ cells in the CP and, like Tead1 and Tead3, Satb2^+^ cells accumulated in the SVZ/IZ (Fig. S4k,l). Together, we observed that Tead1 and Tead3 overexpression show similar phenotypic changes in neuron production while Tead2 induces reciprocal effects on cortical neuron distribution (Table S2). These observations highlight the array of phenotypes induced by Tead TF overexpression and indicate potential different underlying molecular mechanisms downstream of Tead TFs. Interestingly, the Tead2 overexpression recapitulates the Yap1 overexpression phenotypes more closely than Tead1 or Tead3 and suggest a potential cooperation between Yap1 and Tead2 in cortical NSCs.

### Dominant-negative DNA-binding mutant Teads show reciprocal phenotypes *in vivo*

**Figure 4:**
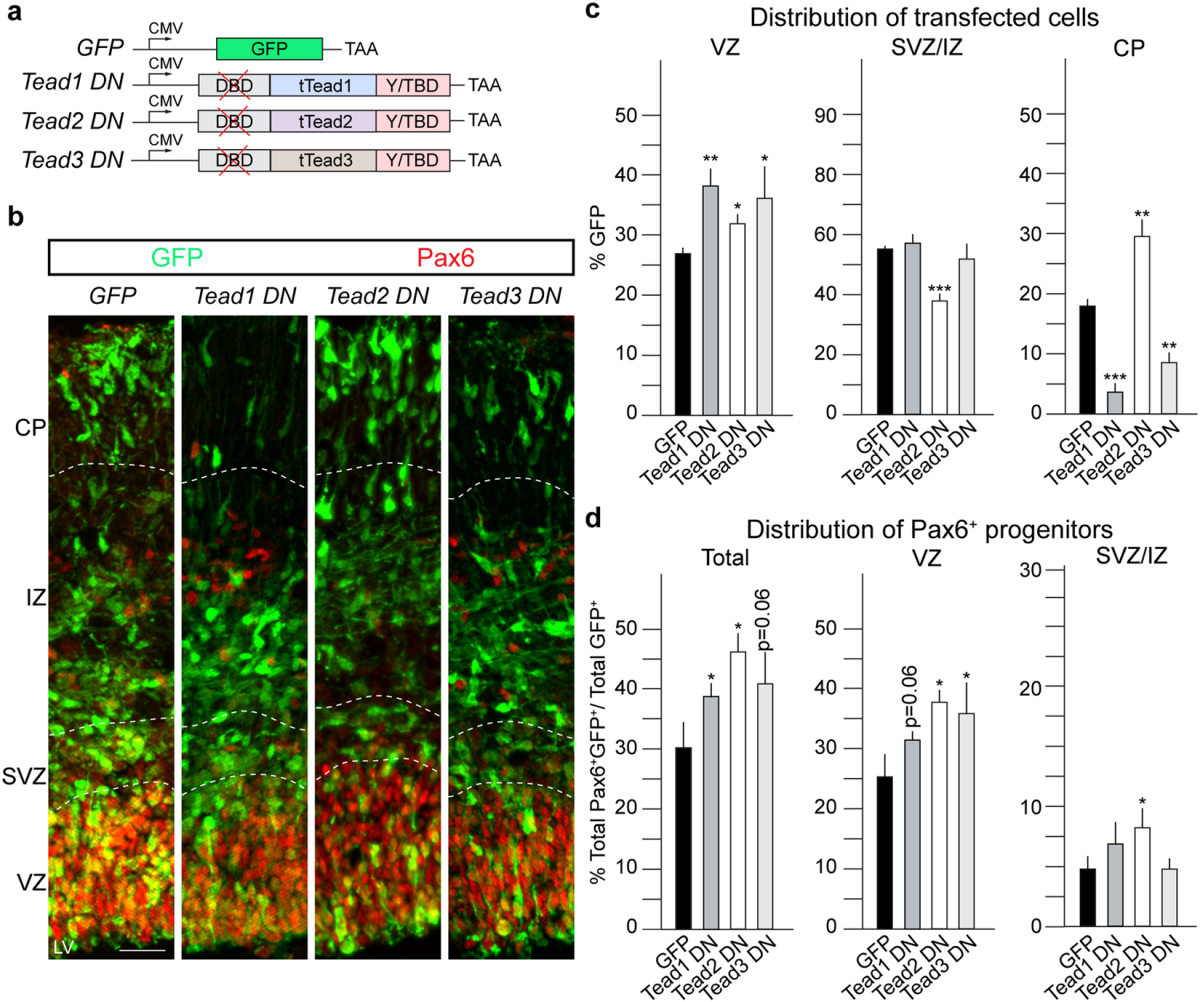
Dominant negative forms of Tead1, Tead2 and Tead3 show reciprocal phenotypes to their corresponding overexpression. (**a**) Experimental paradigm used to perform loss of function of Tead1, Tead2 and Tead3 and empty GFP as a control. DN constructs were cloned without the DNA-binding domains. IUE were performed at E13.5 and brains isolated at E15.5, after 48 hours. (**b**) Coronal sections of transfected cortices immunostained for GFP and Pax6. (**c**) Quantification of distribution of GFP^+^ transfected cells shows Tead1 DN and Tead3 DN induce similar phenotypic changes in cell distribution while Tead2 DN shows an opposite phenotype. (**d**) Quantification of Pax6^+^GFP^+^ cells shows an increase in total Pax6^+^ upon Tead1 DN, Tead2 DN and Tead3 DN, compared to GFP control in VZ and total. DN - Dominant negative, tTead = Truncated Tead, Y/TBD = Yap1/Taz binding domain. Also see Fig. S5. Summaries of the quantifications are in Table S3. Scale bar = 50 μm. Data are shown as average ± SEM, *p = 0.05, **p = 0.01, ***p = 0.001.

To further characterize the potential different roles of Teads in NSCs, we performed loss of function experiments by knockdown with shRNA constructs. As Tead1 and Tead3 induced similar phenotypes, we focused our comparison to knockdown of Tead1 and Tead2 mRNAs. We expressed shRNAs in NSCs and their progeny by IUE, isolated the transfected cells 48 hours post-transfection, and performed RT-qPCR to test the efficiency of target knockdown. However, none of the 10 shRNA constructs induced a significant reduction in Tead1 or Tead2 mRNAs (data not shown). To circumvent this, we generated comparable dominant negative (DN) forms of Tead1, Tead2 and Tead3 by deleting their DNA-binding domains and expressed these *in vivo* by IUE^28^. Although these mutant Tead factors cannot bind DNA, they retain an intact Yap1/Taz binding domain (Fig. 4a). Tead1 DN and Tead3 DN significantly increased retention of cells in the VZ and showed a reduced migration to the CP (Fig. 4b,c). In contrast, Tead2 DN expression increased cells in the VZ and in the CP but resulted in a marked decrease in cells within the SVZ/IZ (Fig. 4b,c). Thus, overexpression of wild type and DN forms of the Teads induced molecule-specific reciprocal phenotypes supporting the validity of the strategy as a loss of function paradigm.

### Tead DNs alter cell fate choice *in vivo*

We examined the effects of the Tead DNs on cell fate. Tead1 DN and Tead3 DN induced increases in the proportion of cells that remained Pax6^+^ (Fig. 4b,d). The DN expressing Pax6^+^ cells were mainly located in the VZ and, in the case of Tead2 DN, were also increased in the SVZ/IZ (Fig. 4b,d). Tead1 DN decreased the total production of Tbr1 cells whereas Tead2 DN and Tead3 DN increased Tbr1^+^ neuron formation, particularly in the CP and SVZ/IZ, respectively (Fig. S5a,b). Conversely, Tead1 DN and Tead3 DN dramatically reduced Tbr1^+^ cells in the CP (Fig. S5a,b). These findings are complementary to the overexpression of the wild type Tead1 and Tead3 (Fig. S4g,h). Therefore, we addressed the expression of Ctip2 in response to Tead DN expression. Whereas Tead1 DN and Tead3 DN reduced Ctip2 expressing cells in the CP, Tead2 DN increased Ctip2^+^ cells in the CP (Fig. S5c,d). Thus, the effects of the Tead1 DN and Tead3 DN on Ctip2 expression were opposite to overexpression of both of their corresponding wild type proteins and the expression of Tead2 DN (Fig. S5c,d compared to Fig. S4c,d and Table S3).

We then addressed potential effects of the Tead DNs on upper layer neuron fate. Tead1 DN and Tead3 DN expression decreased the number of Satb2^+^ neurons in the CP without affecting the total Satb2^+^ cell numbers (Fig. S5e,f). The reduction in Satb2^+^ neurons in the CP was accompanied by an increase in Satb2^+^ cells in the SVZ/IZ suggesting a defect in radial migration. Expression of Tead2 DN did not alter formation or localization of Satb2^+^ neurons (Fig. S5e,f).

### Transactive forms of Tead1 and Tead2 bind common targets

**Figure 5:**
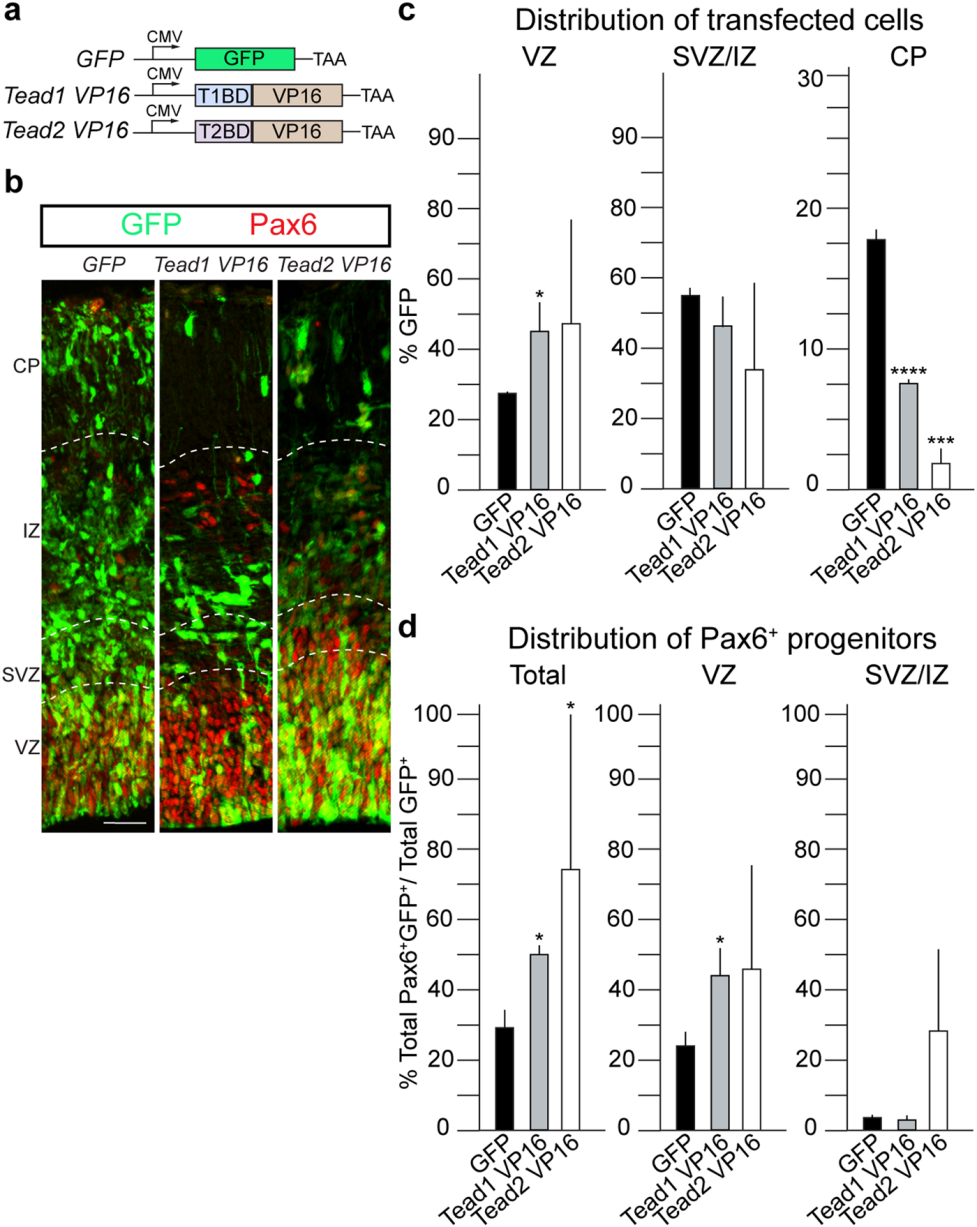
Transactive forms of Tead1 and Tead2 show similar phenotypic changes. (**a**) IUE with the transactive forms (with VP16 domain) of Tead1 and Tead2 were performed at E13.5 and brains isolated at E15.5, after 48 hours. (**b**) Coronal sections of transfected cortices immunostained for GFP and Pax6. (**c**) Quantification of distribution of GFP^+^ transfected cells shows Tead1 VP16 and Tead2 VP16 induce similar phenotypic changes in cell distribution. (**d**) Quantification of Pax6^+^GFP^+^ cells shows an increase in total Pax6^+^ cells upon Tead1 VP16, Tead2 VP16 compared to GFP control in all zones. Summaries of the quantifications are in Table S4. Scale bar = 50 μm. Data are shown as average ± SEM, *p = 0.05, ***p = 0.001, ****p = 0.0001.

The gain of function and DN Timm Maier, Alex Schier, and Tilman Schirmer Biozentrum, University of Basel | Klingelbergstrasse 50/70|CH–4056 Basel experiments indicated that Tead1 and Tead2 can have distinct functions during cortical development. To address whether the different phenotypes induced by Tead1 and Tead2 manipulation are due to selectivity in their DNA-binding domains, we utilized transactive forms of Tead1 and Tead2. We fused the DNA-binding domains of Tead1 or Tead2, without co-activator binding domains, to the viral VP16 transactivation domain and expressed these in NSCs by IUE (Fig. 5a). Hence, gene activation from these constructs is specified purely by binding of the DNA binding domains to sequences in target promoters and not via differences in protein complex formation. Tead1 VP16 and Tead2 VP16 both reduced cell migration to the CP (Fig. 5b,c). This was accompanied by a significant increase in total Pax6^+^ cells compared to controls (Fig. 5b,d and Table S4). Thus, the phenotypic differences we observed in the Tead1 and Tead2 overexpression are not caused by potential differences in the interactions of their DNA-binding domains with target genes.

### Tead1 and Tead2 are predicted to regulate the same targets

**Figure 6:**
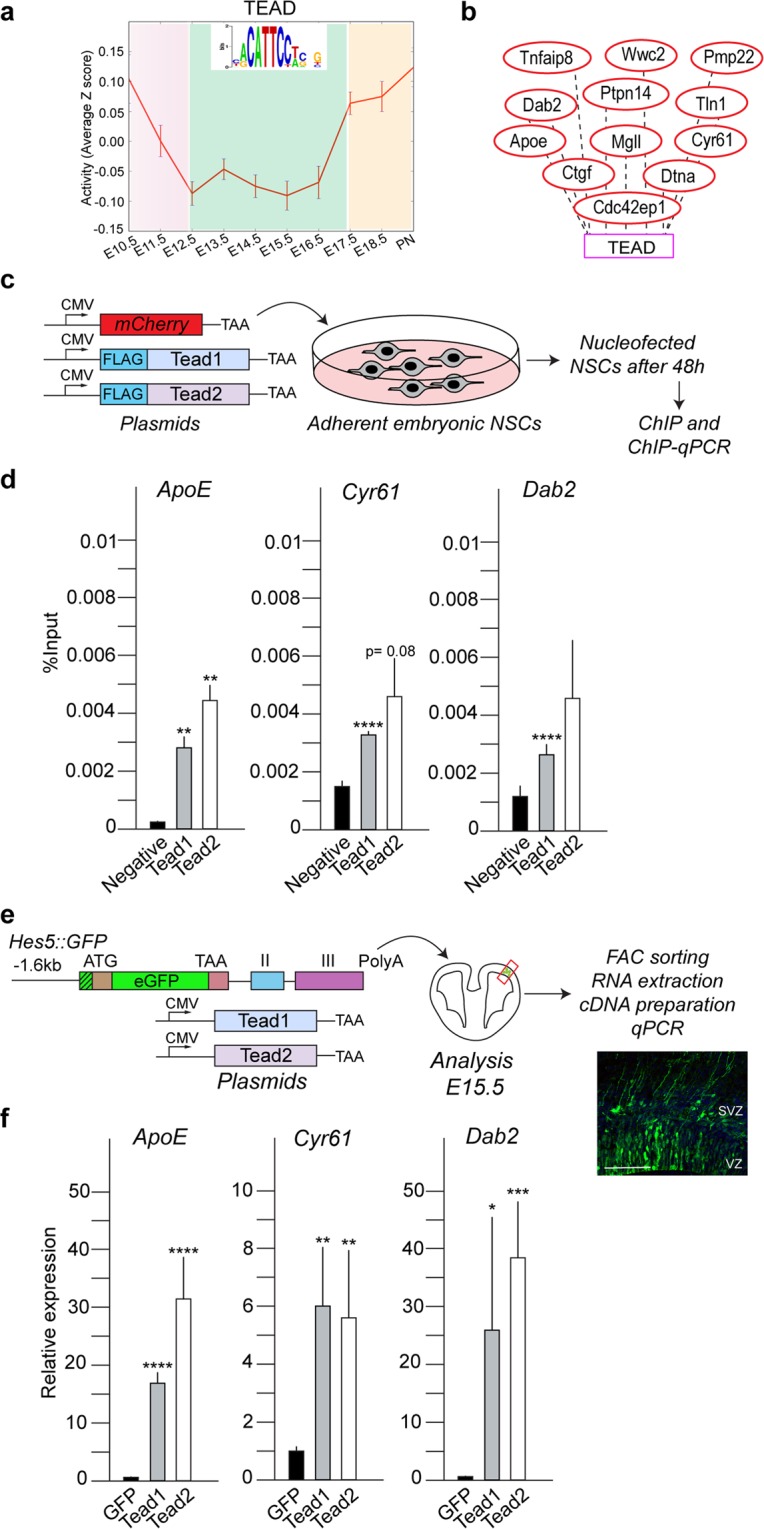
*In silico* predicted Tead targets by ISMARA and their experimental validation. (**a**) Activity of Tead binding motif in NSCs during expansion, neurogenesis and gliogenesis. (**b**) Examples of *in silico* predicted targets of Tead. (**c**) Chromatin Immunoprecipitation for flag tagged-Tead1 and Tead2, performed in adherent NSCs, 48 hours after nucleofection. (**d**) ChIP-qPCR reproducibly pulls-down *ApoE*, *Cyr61* and *Dab2* with both Tead1 and Tead2. An empty mCherry vector was used as the negative control. (**e**) IUE performed with co-transfection of *pBluescript-Hes5::GFP* plasmid, with Tead1 and Tead2 expression constructs, at E13.5. specifically expressed in NSCs in VZ and this approach allows to isolate only transfected NSCs after 48 hours. (**f**) Relative expression of *ApoE*, *Cyr61* and *Dab2* mRNAs show an induced expression upon overexpression of both Tead1 and Tead2. Also see Fig. S6. Summaries of the quantifications are in Table S5. Scale bar = 100 μm. Data are shown as average ± SEM, *p = 0.05, **p = 0.01, ***p = 0.001, ****p = 0.0001.

In order to try to identify the effectors of the Tead TFs in NSCs and explain their effects on differentiation and migration, we performed *in silico* analysis of putative Tead target genes. ISMARA predicted dynamic activity of the Tead binding motif in NSCs from E10.5 to PN during cortical development (Fig. 6a)^23,29^. In addition, ISMARA also predicted a number of likely Tead target genes in NSCs (Fig. 6b, Extended Data Sheet – ISMARA Tead targets and Gene Ontology analysis tabs). We validated some of the predicted Tead target genes by chromatin immunoprecipitation (ChIP) (Fig. S6a,b). We performed ChIP-qPCR for Tead1 and Tead2 from cultured NSCs. In order to perform comparable ChIP, we expressed flag-tagged Tead1 or Tead2 in cortical NSCs and performed ChIP-qPCR for the predicted target genes and Tead motifs (Figs. 6c and S6a,b). *ApoE*, *Cyr61*, and *Dab2* were significantly enriched in both the Tead1 and Tead2 ChIP experiments (Fig. 6d and Table S5A). Although the levels of expression of Tead1 and Tead2 were comparable, the *ApoE* promoter was enriched almost two-fold in the Tead2 compared to the Tead1 ChIP. Together, *in silico* analysis predicted *ApoE*, *Cyr61* and *Dab2* as Tead targets and the promoters of these genes in NSCs were all bound by both Tead1 and Tead2.

In order to test the *in vivo* regulation of *ApoE*, *Cyr61* and *Dab2* by Tead1 and Tead2, we analyzed their expression by NSCs following Tead1 and Tead2 overexpression. We expressed Tead1 or Tead2 by IUE *in vivo* together with a *Hes5::GFP* construct to sort NSCs (Fig. 6e)^25^. 48 hours post-IUE, we sorted the Tead1 or Tead2 expressing *Hes5::GFP*^+^ NSCs by FACS, and performed RT-qPCR for *ApoE*, *Cyr61* and *Dab2* mRNAs as well as other predicted Tead targets (Figs. 6e and S6b). Both Tead1 and Tead2 induced *ApoE*, *Cyr61* and *Dab2* expression in NSCs (Fig. 6f and Table S5). However, in support of the ChIP data, Tead2 expression resulted in a greater induction in *ApoE* mRNA levels than Tead1.

### ApoE, Cyr61 and Dab2 overexpression in NSCs partially recapitulate Tead phenotypes

**Figure 7:**
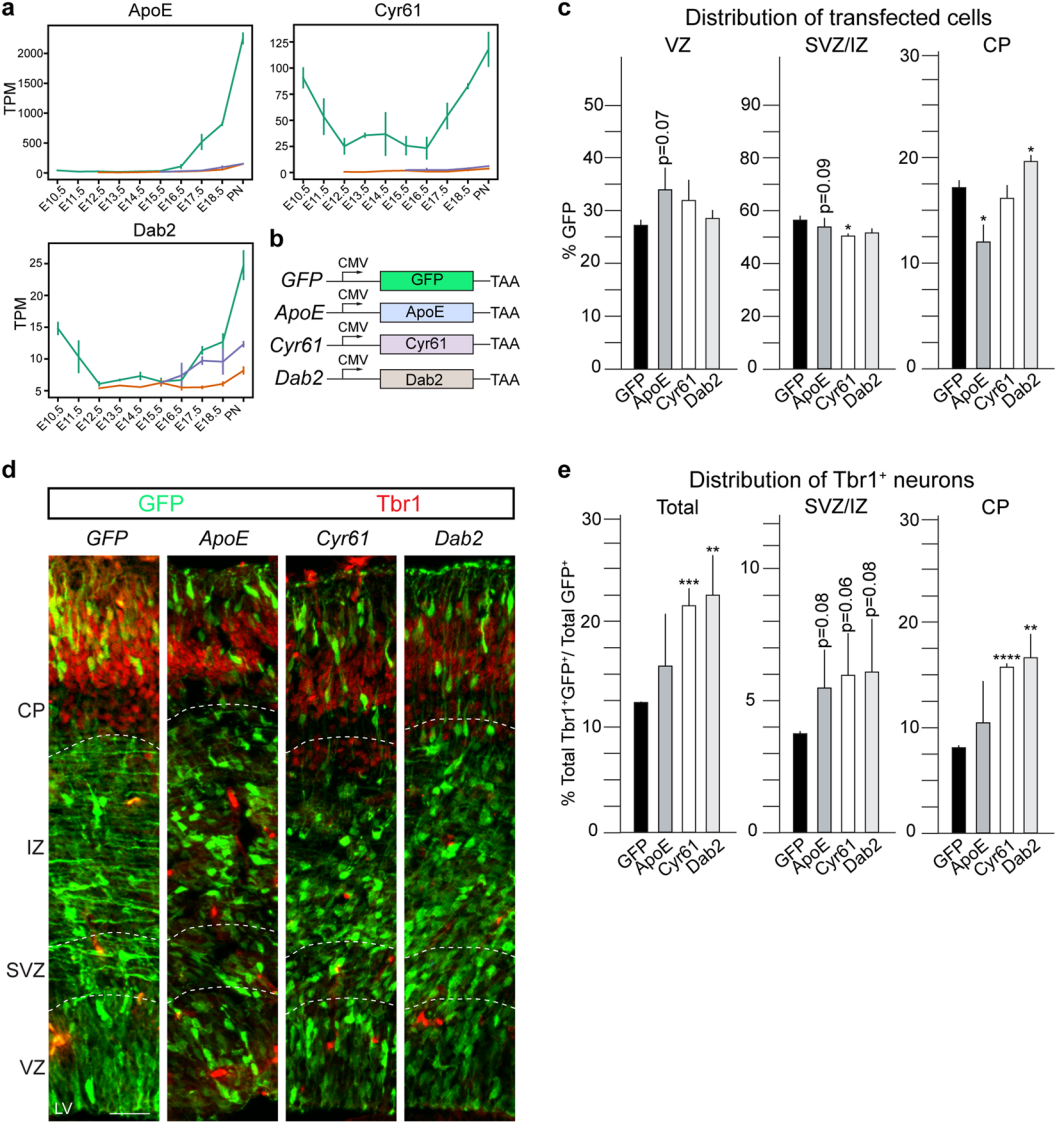
ApoE, Cyr61 and Dab2 overexpression recapitulate Tead overexpression phenotypic changes. (**a**) mRNA expression profiles of *ApoE*, *Cyr61* and *Dab2* in NSCs, BPs and NBNs. (**b**) Expression constructs used for overexpression. (**c**) Quantification of distribution of GFP^+^ transfected cells shows ApoE overexpression recapitulates Tead2 overexpression phenotype while Dab2 overexpression recapitulates the Tead1 overexpression phenotype. (**d**) Coronal sections of transfected cortices immunostained for GFP and Tbr1. (**e**) Quantification of Tbr1^+^GFP^+^ cells shows an increase upon overexpression of Cyr61 and Dab2, compared to GFP control in CP and all zones. Also see Figs. S7 and S8. Summaries of the quantifications are in Table S6. Scale bar = 50 μm. Data are shown as average ± SEM,*p = 0.05, **p = 0.01, ***p = 0.001, ****p = 0.0001.

To confirm that *ApoE*, *Cyr61* and *Dab2* could be potential Tead targets *in vivo*, we analyzed their expression profiles during cortical development (Fig. 7a). *ApoE* is expressed predominantly by NSCs, initially at lower levels during the expansion and neurogenesis periods, and at increasing levels during gliogenesis (Fig. 7a). *Cyr61* is expressed by NSCs throughout cortical development (Fig. 7a), and *Dab2* expression by NSCs reduces dramatically during neurogenesis and increases rapidly at the onset of gliogenesis (Fig. 7a). Therefore, we analyzed the effects of overexpression of ApoE, Cyr61 and Dab2 in NSCs during cortical development by IUE (Fig. 7b). ApoE overexpression at E13.5, a point where its expression is very low, reduced cell migration to the CP with a reciprocal trend for cells to remain in the VZ and a significant increase in Pax6^+^ cells (Fig. S7a,b). These effects partially recapitulated the Tead2 overexpression phenotypes we observed (Fig. 7c compared to Fig. 3c). Cyr61 overexpression resulted in a slight decrease in cells in the SVZ/IZ (Fig. 7c), compared to an increase in cells in the CP caused by Dab2 overexpression (Dab2; 20.5 ± 0.4% compared to 17.8 ± 0.6% in controls), which partially recapitulated the overexpression of Tead1.

Therefore, we addressed whether ApoE, Cyr61 or Dab2 expression changed the fate of NSCs during cortical development. Overexpression of Cyr61 and Dab2 resulted in an increase in Tbr1^+^ neurons, particularly in the CP (Fig. 7d,e). Conversely, ApoE overexpression did not change Tbr1 neuron production (Fig. 7d,e), but significantly increased Ctip2^+^ cells in SVZ/IZ. Cry61 and Dab2 overexpression increased the total number of Ctip2^+^ neurons (Fig. S7c,d). Like ApoE, Cry61 overexpression resulted in an increase in Ctip2^+^ cells in the SVZ/IZ, which was less obvious with Dab2 overexpression (Fig. S7c,d). Although ApoE, Cyr61 and Dab2 did not affect differentiation to Satb2^+^ neurons, ApoE overexpression decreased Satb2^+^ cells in the CP and Dab2 increased Satb2^+^ cells in the SVZ/IZ, likely due to the effects of both of these factors on migration (Fig. S7e,f and Table S6). Thus, ApoE overexpression partially recapitulated some of the Tead2 overexpression phenotypes and Dab2 overexpression recapitulated Tead1 induced phenotypic changes (Fig. 3a,b).

### Tead2 preferentially binds the co-activator Yap1

In order to characterize the potential molecular mechanism underlying the differential phenotypes resulting from Tead1 and Tead2 overexpression, we tested differences in Tead1 and Tead2 binding to Yap1 and Taz. As Yap1 and Taz overexpression phenotypes were similar to Tead2 overexpression, we hypothesized that Yap1/Taz may function preferentially through Tead2 (Fig. S8a). We expressed Tead1-flag or Tead2-flag with either HA-tagged Yap1 or HA-tagged Taz in neuroblastoma cells (N2A) and performed co-immunoprecipitation experiments (Fig. S8b). After normalization to the relative Tead1 and Tead2 input and precipitation levels, Yap1 was found to coprecipitated 2.13-fold and Taz 13-fold more efficiently with Tead2 than with Tead1 (Fig. S8c,d). These results suggest that Yap1 and Taz preferentially bind Tead2 in an overexpression paradigm in N2As and could explain the similarities in the effects seen in the Yap1, Taz and Tead2 overexpression experiments *in vivo*.

## Discussion

Development of the cerebral cortex is precisely controlled. Although some species-specific differences in structure are evident, the isocortex of the cerebral cortex is remarkably similar across mammalian species. However, the process of generating the 6 neuronal layers of the isocortex remains unclear. We addressed gene regulation during cortical development using mouse as a model and found temporal changes in the expression of Hippo signaling components by NSCs, BPs and NBNs. Therefore, we postulated that Hippo signal effectors could regulate different aspects of cortical development in a temporospatial fashion. Intracellular Hippo signaling is controlled by the activity of different surface receptors which regulate the serine threonine kinases Mst1/2 and Lats1/2. Lats1/2 phosphorylate Yap1 and Taz promoting their retention in the cytoplasm and subsequent degradation (Fig. S1a)^8–10^.

Due to the dynamic expression of Hippo signaling components we observed during cortical development, we performed a series of experiments to comparatively examine their roles in the developing brain. We found that the Hippo effectors Yap1 and Taz are major regulators of neurogenesis by promoting NSC maintenance and inhibiting their differentiation. This is supported by previous observations that Yap1 regulates neural tube integrity and induces Cyclin D1 (*Ccnd1*) expression by NSCs thereby promoting proliferation^26,27^. Yap1 has previously been linked to Nf2 activity during progenitor cell activation and formation of the corpus callosum^30,31^. In addition, Yap1 also controls BP proliferation in the developing cortex of ferrets and humans, thus, contributing to the evolutionary neocortical expansion^32^. The block of differentiation and exit from the VZ we observed here are reminiscent of preventing epithelial to mesenchymal transition (EMT). Scratch1/2 and Lzts1 have been shown to regulate progenitor exit from the VZ and control differentiation in an EMT-like process^33,34^. It will be interesting to address a potential interaction between YAP/Taz and EMT control in the VZ. Although NSCs express higher levels of *Yap1* than *Taz*, overexpression experiments show that both Hippo co-activators can induce similar downstream functions.

The importance of Hippo signaling in cortical development was exemplified by analysis of Fat4 and Dchs1 functions. Fat4 and Dchs1 are receptor and ligand of the Hippo pathway, and mutations in *FAT4* and *DCHS1 *cause Van Maldergem syndrome in humans. When Hippo signaling receptors are inactive, Yap1 and Taz escape degradation, translocate to the nucleus, and interact with TFs. Therefore, inactivation of Fat4 and Dchs1 results in stabilization of Yap1 protein and activation of Hippo signaling. Fat4 and Dchs1 loss of function in mouse NSCs induced phenotypes resembling Van Maldergem syndrome with the formation of periventricular heterotopias^13,14^. Periventricular heterotopias are aggregations of misplaced neurons in the lining of the cerebral ventricles. In the Hippo pathway, Yap1 and Taz interact with and regulate the Tead TFs^8,9,35,36^. The functions of Tead TFs have been extensively studied during heart morphogenesis, vasculogenesis, muscle development, EMT and in various cancers, however, their functions during cortical development have not been elucidated^6,11,12,28,37^. Previous analyses in other systems proposed that Tead TFs are redundant and overlapping^21^. Surprisingly, we found that Teads often play reciprocal roles in corticogenesis by differentially regulating NSCs maintenance, proliferation and differentiation as well as neuroblast migration.

Previous *in silico* analysis suggested that Tead4 is a potential mediator of the Yap1/Taz effects in neural progenitors^38^. Our expression analysis shows that Tead4 is not expressed to detectable levels by cortical NSCs, and that Yap1 preferentially works through Tead2 to regulate NSC maintenance and differentiation during the peak period of neurogenesis in the dorsal cerebral cortex. As Yap1, Taz and Tead2 expression block migration of cells to the CP and, increase progenitors in the germinal zones, it is possible that the hyper-expansion of neural progenitors and miss-migration of immature neurons in Van Maldergem syndrome is caused, at least in part, by aberrant Tead2-induced transcription. In addition, Pard3 has been demonstrated to play a role in cortical size control in conjunction with Yap1 and Taz^39^. Aberrant activity of Pard3 is also implicated in heterotopias and increased susceptibility to seizures^39^.

How Hippo signaling regulates the migration of progenitors and immature neurons from the germinal zones of the VZ and SVZ to the CP was unclear. We identified *ApoE* and *Dab2* as novel targets of the Teads and the Hippo pathway. *ApoE* and *Dab2* are expressed by NSCs at low levels during expansion and neurogenesis and increase during gliogenesis, reaffirming the role of ApoE in astrogliogenesis during later stages of corticogenesis^40^. ApoE is a ligand for ApoER2, a Reelin signaling receptor and regulator of migration of neuroblasts in the developing brain^41–44^. Dab2 is an intracellular adapter protein that regulates ApoER2 signaling. Reelin is expressed by Cajal-Retzius cells in the marginal zone of the CP forming an outside-in gradient that supports radial glial fiber integrity, directed immature neuronal migration and cortical layering. When expressed in cortical progenitors *in vivo*, ApoE partially recapitulated the migratory phenotype observed following forced Tead2 expression. We propose that the increase in ApoE expression in the VZ and SVZ at E13.5, by activation of Tead2 or overexpression of ApoE itself, results in an inside-out gradient of this second Reelin signaling ligand. As we did not observe disruption of the radial fiber network following Tead2 or ApoE expression, we hypothesize that ApoE overexpression destroys the normal directed migration trajectory of the immature neurons by counteracting the normal Reelin gradient. Hence, our findings provide a potential mechanistic link between Hippo signaling and the Reeling pathway, a known regulator of cortical development.

We also identified Cyr61 as a Tead target in NSCs. *Cyr61* is expressed throughout cortical development and its mRNA levels reduce during neurogenesis. Cyr61 is a component of Integrin and canonical Wnt signaling pathways, which are also involved in NSC maintenance differentiation and neuronal migration^45^. *Cyr61* has been described as a Yap1 target in the skin^46^. It will be of interest in the future to address whether Hippo/Tead signaling act as a modulator and transistor to control the interface and outputs of different pathways (Reelin, Wnt and Integrin) to fine-tune cell fate during cortical development.

Hippo signaling is regulated though a cascade of kinases including Mats1/2 and Lats1/2. Inhibition of Lats1/2 in mouse cortical progenitors results in stage specific changes in proliferation, cell death and disruption of cortical formation in a Yap1/Taz-dependent fashion^38^. We did not observe the same massive cell death in any of our overexpression experiments. One explanation could be that blocking the Lats1/2 kinases results in global activation of the Hippo cascade whereas our experiments focally altered specific parts of the pathway. Alternatively, Lats1/2 and Yap1/Taz may regulate other signaling pathways independent of Tead TFs and these parallel pathways contribute to the induction of apoptosis seen following blockade of Lats1/2. Yap1/Taz are known to regulate transcription though Smads downstream of TGFβ signaling and Runx TFs^8–10^. In fact, Lats1/2 inhibition resulted in Trp53-independent global hyper-transcription and mitotic stress indicating convergence with other cellular stimuli^38^. Interestingly, Teads also interact with the coactivator Vgll4 which competes with and inhibits Yap1 function^47^. We found that Vgll4 is expressed by NSC, BPs and NBNs, and thus may modulate Hippo signaling upstream of Teads in a cellular context dependent manner (Fig. S2b). Future experiments will be required to elucidate the cross-talk and dynamic interaction mechanisms between Hippo/Tead and other signaling pathways during corticogenesis.

Our findings indicate that Tead TF functions are specific and dynamic during cortical development. Differential signaling though the Teads can result in different cellular responses. However, we also found that the upstream components of the Hippo cascade show developmentally regulated, and cell-type specific expression patterns. Mst1/2 and Lats1/2 kinases are in complexes with regulatory proteins including Sav1, Mob1a, Mob1b, Wwc1, Wwc2, Nf2, 14-3-3ε, Cdc73, and Amot, and the genes encoding these protiens are all dynamically expressed by NSCs, BPs and NBNs (Fig. S2b)^48^. Interestingly, mutations in *Ywhae*, which encodes 14-3-3ε protein, result in cortical defects and aberrant neuroblast migration through miss-regulation of NUDEL, a Lis1-binding protein. In humans, *YWHAE* mutations also segregate with and contribute to Miller-Dieker syndrome, a characteristic of which is the formation of periventricular heterotopias^49^. Whether the role of 14-3-3ε in the regulation of Yap1 and Hippo signaling also contributes to Van Maldergem syndrome is not clear. It will be interesting in the future to address the functions and cross-talk between Hippo regulatory proteins and other pathways and to elucidate whether the Tead TF targets are miss-expressed in patients with Van Maldergem syndrome or other diseases of the human nervous system.

## Methods

**Table of reagents**

**Table:** 

**Reagent or Resource**		
**Antibodies**	**Source**	**Identifier**
Rabbit anti-Caspase 3 (1:500)	Cell Signaling Technology	Cat# ab 9664 RRID: AB_2070042
Rat anti-Ctip2 (1:500)	Abcam	Cat# ab18465, RRID: AB_2064130
Mouse anti-Flag (1:1000)	Sigma	Cat# F3165; RRID: AB_259529
Mouse anti-Gapdh (1:1500)	Calbiochem	Cat# CB1001, RRID: AB_2107426
Sheep anti-GFP (1:250)	AbD Serotec/Biorad	Cat# 4745-1051, RRID: AB_619712
Rabbit anti-HA tag (1:1000)	Cell Signaling Technology	Cat# 3724, RRID: AB_1549585
Rabbit anti-pHH3 (1:500)	Millipore	Cat# ab 06-570 RRID: AB_310177
Rabbit anti-Ki67 (1:500)	Abcam	Cat# ab 15580 RRID: AB_443209
Rabbit anti-Pax6 (1:500)	Covance	Cat# PRB-278P, RRID: AB_291612
Mouse anti-Satb2 (1:200)	Abcam	Cat# ab51502, RRID: AB_882455
Rabbit anti-Tbr1 (1:500)	Abcam	Cat# ab31940, RRID: AB_2200219
Rabbit anti-Tbr2 (1:400)	eBioscience	Cat# ab 14-4875-82, RRID: AB_11042577
Mouse anti-Tead1 (1:200)	Santa Cruz	Cat# sc-376113 RRID: AB_10988229
Rabbit anti-YAP (1:100)	Cell Signaling Technology	Cat# 14074 RRID: AB_2650491
Donkey anti-Sheep, Alexa 488 (1:500)	Jackson ImmunoResearch Labs	Cat# 713-545-147, RRID: AB_2340745
Donkey anti-Rabbit, Cy3 (1:500)	Jackson ImmunoResearch Labs	Cat# 711-165-152, RRID: AB_2307443
Donkey anti-Mouse, Cy3 (1:500)	Jackson ImmunoResearch Labs	Cat# 715-165-151, RRID: AB_2315777
Donkey anti-Rat, Cy3 (1:500)	Jackson ImmunoResearch Labs	Cat# 712-166-153, RRID: AB_2340669
Donkey anti-Mouse, HRP (1:10000)	Jackson ImmunoResearch Labs	Cat# 715-035-151, RRID: AB_2340771
Donkey anti-Rabbit, HRP (1:10000)	Jackson ImmunoResearch Labs	Cat# 711-035-152, RRID: AB_10015282
**Chemicals**	**Source**	**Identifier**
16% Formaldehyde Solution (w/v), methanol-free	Sigma	28908
DNase I, RNase-free	Sigma	04716728001
DNase I Grade II	Roche	10104159001
Glycine	Sigma	50046-1KG
L_Cysteine	Sigma	168149
Papain	Sigma	P3125-100MG
Trypsin inhibitor from Glycine max (soybean)	Sigma	T6522-5x100MG
L15 Medium	Invitrogen	31415029 (31415086)
PBS cell culture	Dulbecco	14080089 (14080048)
Transfectin	BioRad	1703351
Complete Proteinase Inhibitor Cocktail	Roche	11697498001
PMSF	Sigma	P7626 (78830)
SensiFast SYBR Kit	Bioline	BIO-02005
Triton X-100	Fisher	BPE151-500
TRIzol	Invitrogen	VX15596018
Dynabeads	Invitrogen	10765583
Glycoblue Co-precipitate	Life Technologies	D1417005
Bioscript, Reverse transcriptase	Bioline	BIO-27036-4
Transfectin Reagent	BioRad	1703352
Fastgreen	Sigma	F7252
P3 primary cell 4D-Nucleofector X kit 24 reactions	Lonza	LZ-V4XP-3024
Poly L- Lysine hydrobromide	Sigma	P9155-5MG
Laminin	Sigma	L2020-1MG
Phenol-chloroform isoamyl alcohol	Life Technologies	15593-031
B27 supplement+A26	Gibco	17504-044
Beta-mercaptoethanol	Sigma	M6250-100ML
DMEM/F12	Gibco	31966-047
DMEM (high glucose)	PAN Biotech	P04-04510
FBS	PAA	A15-101
Nitrocellulose membrane	Protan, GE	Z670995-1EA
**Experimental models**	**Source**	**Identifier**
Mouse: *Hes5::GFP*	Verdon Taylor (Basak *et al*., 2007)	Tg(Hes5-EGFP)2Vtlr
Mouse: *Tbr2::GFP (*Eomes^tm2.1Rob^)	MGI	Cat# 4399136, RRID: MGI: 4399136
Mouse: C57BL/6J	Janvier Labs	RRID: IMSR_JAX: 000664
Neuroblastoma cells (N2A)	ATCC	Cat# CCL-131 RRID: CVCL_0470
Wt Neurospheres	Derived from C57Bl/6J embryos	N/A
Wt adherent neural stem cells	Derived from C57Bl/6J embryos	N/A
**Recombinant DNA**	**Source**	**Identifier**
pMYs-EGFP	Diepenbruck *et al*., 2014	N/A
pCMV-Flag-Tead1	This paper	N/A
pCMV-Flag-Tead2	RIKEN Bioresource	Cat# RDB12171
pCMV-Flag-Tead3	This paper	N/A
pCMV-Flag-ApoE	This paper	N/A
pCMV-Flag-Cyr61	This paper	N/A
pCMV-Flag-Dab2	This paper	N/A
pMys-HA-Yap1-IRES-GFP	Diepenbruck *et al*., 2014	N/A
pMys-HA-Taz-IRES-GFP	Diepenbruck *et al*., 2014	N/A
pMYs-HA-Tead2FL-IRES-EGFP	RIKEN Bioresource	Cat# RDB12173
pMYs-HA-Tead1-VP16-IRES-EGFP	RIKEN Bioresource	Cat# RDB12172
pMYs-HA-Tead2-VP16-IRES-EGFP	RIKEN Bioresource	Cat# RDB12174
pCMV-Flag-Tead1-Dominant negative	This paper	(nucleotide 579-1800)
pCMV-Flag-Tead2-Dominant negative	This paper	(nucleotide 420-1421)
pCMV-Flag-Tead3-Dominant negative	This paper	(nucleotide 441-1571)
pBluescript-*Hes5::GFP*	Basak *et al*., 2007	N/A
**Oligonucleotides**	**Source**	**Identifier**
ApoE_Forward_5′-CTGACAGGATGCCTAGCCG-3′	https://pga.mgh.harvard.edu/primerbank/	N/A
ApoE_Reverse_5′-CGCAGGTAATCCCAGAAGC-3′	https://pga.mgh.harvard.edu/primerbank/	N/A
ApoE_Forward_5′-GAGTTCGCTATCTCGGCACC-3′	This paper	N/A
ApoE_Reverse_5′-TGGAAAGCAGGACTTAGCCG-3′	This paper	N/A
ApoE_Forward_5′-CGCTCTTCCCAAAGGTCTGT-3′	This paper	N/A
ApoE_Reverse_5′-TGGAAAGCAGGACTTAGCCG-3′	This paper	N/A
ApoE_Forward_5′-CGCTGCCAAAAATTCCAGCT-3′	This paper	N/A
ApoE_Forward_5′-GTACCACTTCGCAGGGATGG-3′	This paper	N/A
ApoE_Reverse_Xba1_5′-ATCTCTAGATCATTGATTCTCCTGGGCCAC-3′	This paper	N/A
Beta-actin_Forward_5′-AGGTGACAGCATTGCTTCTG-3′	This paper	N/A
Beta-actin_Reverse_5′-GGGAGACCAAAGCCTTCATA-3′	This paper	N/A
Cyr61_Forward_Not1_5′-TTCCGCGGCCGCATGAGCTCCAGCACCTTC-3′	This paper	N/A
Cyr61_Reverse_Xba1_5′-CCCTCTAGATTAGTCCCTGAACTTGTGGAT-3′	This paper	N/A
Dab2_Forward_5′-CCCCTGAACGGTGATACTGAT-3′	This paper	N/A
Dab2_Reverse_5′-AAGTCCTGCTTTACGCCATTC-3′	This paper	N/A
Dab2_1_Forward_5'-TTGGAAGACTCGGCAGACAC-3′	This paper	N/A
Dab2_1_Reverse_5′-GGCCACTCCCGGTAGAGATA-3′	This paper	N/A
Dab2_2_Forward_5′-GGCGCTGGGGAAATCTTACA-3′	This paper	N/A
Dab2_2_Reverse_5′-CCTTGAGTCCGACCCCAAAG-3′	This paper	N/A
Dab2_Forward_Not1_5′-TCGGCGGCCGCATGTCTAACGAAGTAGAAA-3′	This paper	N/A
Dab2_Reverse_Xba1_5'-CCATCTAGACTAGGCAAAAGGATTTCCGAA-3′	This paper	N/A
Gapdh_Forward_5′-CTCCCACTCTTCCACCTTCG-3′	This paper	N/A
Gapdh_Forward_5′-CCACCACCCTGTTGCTGTAG-3′	This paper	N/A
Tead1_Forward_5′-AAGCTGAAGGTAACAAGCATGG-3′	https://pga.mgh.harvard.edu/primerbank/	N/A
Tead1_Reverse_5′-GCTGACGTAGGCTCAAACCC-3′	https://pga.mgh.harvard.edu/primerbank/	N/A
Tead1_Forward_5′-CGCTCGCCAATGTGTGAATA-3'	https://pga.mgh.harvard.edu/primerbank/	N/A
Tead1_Reverse_5′-AATACACAGGCCATGCAGAG-3′	https://pga.mgh.harvard.edu/primerbank/	N/A
Tead1_Forward_5′-TTCGAGAAATTCAAGCCGCC-3′	https://pga.mgh.harvard.edu/primerbank/	N/A
Tead1_Reverse_5′-GAGACGATCTGGGCTGATGA-3'	https://pga.mgh.harvard.edu/primerbank/	N/A
Tead1_Forward_5′-CCCTCAAAACGCCTTCTTCC-3′	https://pga.mgh.harvard.edu/primerbank/	N/A
Tead1_Reverse_5′-AACCTCGCATACTCCGTCTC-3′	https://pga.mgh.harvard.edu/primerbank/	N/A
Tead1_Forward_5′-GACATGCTTGGTTGAACTATCCT-3′	https://pga.mgh.harvard.edu/primerbank/	N/A
Tead1_Reverse-5′-GAGGGGTGATGTCTTCCTCC-3′	https://pga.mgh.harvard.edu/primerbank/	N/A
Tead2_Forward_5′-CCCTCCTTGCTCTTCTGGAA-3′	https://pga.mgh.harvard.edu/primerbank/	N/A
Tead2_Reverse_5′-CCACTTCACCCTACCCCAAG-3′	https://pga.mgh.harvard.edu/primerbank/	N/A
Tead2_Forward_5′-CCTGTCAGATGAGGGCAAGA-3′	https://pga.mgh.harvard.edu/primerbank/	N/A
Tead2_Reverse_5′-ACTTGGTCCTTCAGCTTGGA-3′	https://pga.mgh.harvard.edu/primerbank/	N/A
Tead2_Forward_5′-TCCACATCAGTCAGCAGTGT-3′	https://pga.mgh.harvard.edu/primerbank/	N/A
Tead2_Reverse_5′-ACTTGACGAGGAAGAAGGCA-3′	https://pga.mgh.harvard.edu/primerbank/	N/A
pCMV_Tead2_Forward_HindIII_5′-ACCCAAGCTTCCACCATGGAC-3′	This paper	N/A
pCMV_Tead2_Reverse_XbaI_5′-CGAGCATGCATCTAGAGGG-3′	This paper	N/A
pCMV_Tead2_Reverse_5′-ATCGTCTGGAAGGCCTTGTCCTTGGAGACTTGGTCC-3′	This paper	N/A
Tead2_DN_EcoRI_Forward_5′-TTCAGAATTCATGATTGCCCGTTACATCAA-3′	This paper	N/A
Tead2_DN__XbaI_Reverse_5′-CCTGTCTAGACCTGAGTGTCCCTGTTTGT-3′	This paper	N/A
Tead2_DN_EcoRI_Forward_ 5′-TTCAGAATTCATGTCGAGAGAAATTCAGTCCAAG-3′	This paper	N/A
Tead1_DN_Not1_Forward_5'-TATTCACGCGGCCGCATGGAGCAGAGT-3′	This paper	N/A
Tead1_DN_Xba1_Reverse_5′-GCCGATTCTAGATGTAGATATGGTGCTGTG-3′	This paper	N/A
Tead1_Forward_5′-ACAAGGCCTTCCAGACGATG-3′	https://pga.mgh.harvard.edu/primerbank/	N/A
Tead1_Reverse_5′-TGTGAGAAGGGCTTCACGTC-3′	https://pga.mgh.harvard.edu/primerbank/	N/A
Tead1_transgene_Forward_5′-AAGCGGAGAATTCCACCAGG-3′	This paper	N/A
Tead1_transgene_Reverse_5′-TCCTCACAAGACGTCAAGCC-3′	This paper	N/A
Tead2_transgene_Forward_5′-GCCTCTGACCTACCAGGGTA-3′	This paper	N/A
Tead2_transgene_Reverse_5′-TGCCTCTGGAACGAGTCAAC-3′	This paper	N/A
Tead3_Not1_Forward_5′-GATCGAGCGGCCGCCACTGTGCTGGAT-3′	This paper	N/A
Tead3_Xba1_Reverse-5′-TACATTTCTAGAGAGCTCGGATCCACT-3′	This paper	N/A
Tead3_DN_Not1_Forward_5′-TATCGAGCGGCCGCATGGCATCCATGTCG-3′	This paper	N/A
Yap1_Forward_5′-GCATGAGCAGCTACAGCATC-3′	https://pga.mgh.harvard.edu/primerbank/	
Yap1_Reverse_5′-CCAAGATTTCGGAACTCAGC-3′	https://pga.mgh.harvard.edu/primerbank/	
Yap1_Foward_5′-GGAGACACCATCAGCCAAAG-3′	https://pga.mgh.harvard.edu/primerbank/	
Yap1_Reverse_5′-ACTCCACGTCCAAGATTTCG-3′	https://pga.mgh.harvard.edu/primerbank/	
**Resource**	**Source**	**Identifier**
Fiji	Hosted by University of Wisconsin	https://imagej.net/Fiji/Downloads
Photoshop	Adobe	N/A
Illustrator	Adobe	N/A
Prism 7	GraphPad Software, Inc	https://www.graphpad.com/scientific-software/prism/
R	R Core Team	https://www.r-project.org
*In situ* hybridization data for Tead1	Allen Brain Atlas	http://developingmouse.brain-map.org/gene/show/21437
*In situ* hybridization data for Tead2	Allen Brain Atlas	http://developingmouse.brain-map.org/gene/show/21438
*In situ* hybridization data for Tead3	Allen Brain Atlas	http://developingmouse.brain-map.org/gene/show/21439

### Mice and husbandry

Wild type (Wt), *Hes::GFP*^25^ *and Tbr2::GFP*^24^ transgenic lines have been described previously. Mice were maintained on a 12hr day-night cycle with free access to food and water under specific pathogen-free conditions according to the Swiss Federal regulations and ARRIVE guidelines. All experimental procedures were approved by the Basel Cantonal Veterinary Office and ethics commission and performed under license number 2642 in accordance to 3R guidelines.

### Tissue preparation and fluorescence assisted cell sorting (FACS)

Dorsal cortices from embryonic day (E10.5) to postnatal day 1 (PN) were micro-dissected and dissociated into single cell suspensions using Papain and Ovo-mucoid mix (previously described by Giachino *et al*., 2009)^50^. Cells were washed with L15 medium and FAC-sorted for GFP^+^ NSCs using FACS AriaIII (BD Biosciences). For each time point, 3-4 biological replicates were generated.

### RNA Isolation and RNA-sequencing

Total RNA was isolated from FAC-sorted GFP^+^ cells with *Hes5::GFP* and *Tbr2::GFP* transgenic lines using TRIzol reagent. A time course was performed with NSCs isolated at each time point during development from E10.5 to postnatal day 1 (PN). The integrity of the RNA samples was checked using the Agilent 2100 Bioanalyzer. Their concentration was measured using Quant-IT RiboGreen RNA Assays (Life Technologies). Libraries for Illumina sequencing were prepared with the TruSeq RNA Library Prep Kit v2 (Illumina) and quality checked using the Fragment Analyzer high-sensitivity NGS kit (AATI). SR50 sequencing was performed on Illumina HiSeq2000 and HiSeq2500 systems with v3 and v4 SBS chemistry, respectively. For the samples that are included the read length is 51 for most samples (51 samples) 50 for 13 and 63 for 6 samples (E10.5 and E11.5 NSC). The reads per sample before mapping was between 10 and 70 million reads, mostly samples had between 10 and 20 million reads (Fig. S2a).

### Cloning of dominant-negative (DN) constructs for Tead TFs

Dominant-negative constructs for Tead1 (nucleotide 579-1800), Tead2 (nucleotide 420-1421) and Tead3 (nucleotide 441-1571) were cloned by removing the N-termini, containing the DNA-binding domains.

### IUE for *in vivo* manipulation of NSCs and RNA isolation

Pregnant C57Bl/6 mice at E13.5 were anaesthetized with isoflurane. Their uteri were exposed and DNA expression constructs were microinjected using Pneumatic Pico Pump, (WPI Rnage) and Borosilicate glass capillaries (Kwick-Fil; Hampton Research). The capillaries were pulled in a micropipette puller (Sutter Instrument Co.). The tips of the capillaries were sharpened using a capillary sharpener (Bachofer). The capillaries were loaded with 10μl of the plasmid. Plasmid stocks were prepared using endotoxin-free conditions. Plasmids were dissolved in sterile water at high concentrations (2-5 μg/μl). A fast-green contrast dye was added to the plasmids, to visualize the area of injection in the lateral ventricle. The overexpression or dominant negative constructs were electroporated in a molecular ratio of 3:1, with transfection reporter vector (pMYs-IRES-GFP). Mice were secured on a heated pad to maintain a good body temperature, while being anaesthetized with 1–2% isoflurane (Baxter), along with a constant flow of O_2_. A depilation cream was administered to remove the fur from the abdomen. Throughout the course of the procedure, the embryos and the peritoneal cavity was moistened with sterile HBSS to prevent drying. The uterine horn and the embryos were handled under sterile conditions, by hand and a cold light source was used to illuminate the developing embryos. We injected 1–2 μl of 2 μg/μl DNA solution, into the lateral ventricles (LV) of each embryo. The embryos were electroporated (BTX ECM830, Harvard Apparatus) with five pulses of 50 V and a pulse length of 50 ms at 950 ms intervals. The orientation of the electrodes directs the regions to be transfected. After the injections, the uteri were returned to the abdomen and the muscles, skin sutured. The females were allowed to recover under a heating lamp with constant observation. Postoperative analgesic (Temgesic) was administered. The animals were sacrificed after 48 hours by CO_2_ inhalation. The embryos were isolated and brains dissected out. Positive brains were checked under Fluorescence microscope for GFP reporter and processed for tissue dissociation (as described above) and FACS or prepared for freezing and subsequent sectioning. Alternate positive samples were collected and dissociated as described above. Cells were sorted for GFP and RNA isolated from transfected cells. cDNA was prepared using Bioline Bioscript kit, followed by gene expression analysis using Bioline Sensifast SYBR.

### Tissue preparation and immunohistochemistry

Positive brains isolated and fixed with 4% PFA in 0.1M phosphate buffer, then cryoprotected with 15% and 30% sucrose in phosphate buffer. Brains were embedded and frozen in OCT (TissueTEK) and sectioned 20 μm on slides (Superfrost glass slides, Thermo Scientific) by cryostat (Leica). Sections were dried at room temperature (RT) before antigen retrieval was performed with 1X Citrate buffer, at 80 °C for 15 minutes. Sections were blocked with 5% Normal donkey serum with 0.01% Triton X-100 and 0.1M phosphate buffer for 2h at RT. Sections were incubated overnight at 4 °C with primary antibody solutions made with blocking buffer. Sections were washed with phosphate buffer at RT and incubated with secondary antibody solutions with blocking buffer for 3h at RT. Sections were washed again as above and incubated with 1:1000 DAPI to stain the nuclei. The sections were rinsed once with phosphate buffer and dried at RT. Sections were mounted in mounting media containing diazabicyclo-octane (DABCO; Sigma) as an anti-fading agent and visualized using Zeiss Apotome 2 microscope.

### Adherent NSC culture and Amaxa nucleofection *in vitro*

Primary NSCs were isolated from E13.5 dorsal cortices and cultured in DMEM/F12 + Glutamax medium (with 2% B27 and 20 ng/μl FGF2) as neurospheres (as described by Giachino *et al*., 2009)^50^. The tissue was dissociated as described above. On day 5 of culture, the neurospheres were plated on 100 μg/μl Poly L-Lysine and 1 μg/μl Laminin pre-coated sterile 6-well plates. The culture was continued until confluency was reached and adherent NSCs were passaged and expanded.

Adherent NSCs were transfected with expression constructs following the Amaxa nucleofection kit instructions. Briefly NSCs were detached using trypsin for 5′, followed by incubation with Ovo-mucoid mix. Cells were washed with sterile tissue culture grade phosphate buffer. We performed the nucleofections in 16-well strips and used phosphate buffer for transfections. Expression constructs: 7.5 μg pCMV Flag Tead1; pCMV Flag Tead2 and pCMV mCherry (transfection control) were used for the nucleofections. The cells were kept in culture for 48 hours post-transfection and collected for ChIP assays.

### Chromatin immunoprecipitation (ChIP)

Transfected NSC were fixed with 1% PFA (Sigma) for 8 mins at RT. PFA was quenched with 125 mM Glycine and cells were washed with phosphate buffer with protease inhibitors and PMSF. Cells were lysed with cell lysis buffer and SDS-lysis buffer sequentially. Nuclei were sonicated using Diagenode Biorupter for 30s on and off cycles, 15 times. The supernatant was diluted with ChIP dilution buffer and used for IP using 1 μg of α-Flag antibody (Sigma, F3165). The ChIP protocol followed was the modified Millipore-Merck protocol. We used Protein-G Dynabeads for the pulldown. Beads were washed with low salt, high salt and TE buffer respectively. Fresh elution buffer was used to elute the DNA and reverse cross-linking was performed overnight at 65 °C in high salt conditions. The eluates were treated with Proteinase K and RNase and purified using Phenol-Chloroform iso-amyl alcohol (Invitrogen). From ISMARA, we obtained the putative binding sites of the Teads and we tested the IP eluates for pull-down, using primers directed against these sites. We tested the targets for few of the *in-silico* predicted Tead targets.

### Neuroblastoma (N2A) cell culture and immunoprecipitation (IP)

N2A cells were cultured in DMEM medium with high glucose, FBS and PenStrep. These cells were transfected with expression constructs at 60–70% confluency with Transfectin reagent (BioRad, 1703352). Protein lysates were isolated after 48 hours and processed for immunoprecipitation using α-Flag antibody (Sigma, F3165). Dynabeads were used for the IP and proteins were eluted in Lämmli-buffer containing 2-mercaptoethanol, boiled for 10′. Protein samples were separated using 12% SDS-poly-acrylamide gels and transferred to Nitrocellulose membranes (Protan, GE). Primary antibody α-HA antibody (Cell Signalling, 3724) was incubated with the membrane overnight at 4 °C. Secondary antibody horse radish peroxidase conjugated α-rabbit-Ig (Jackson Immunoresearch Labs, 711-035-152) incubation was performed for 1h at RT. Detection was done by chemiluminescence (ECL, GE Healthcare).

### Quantification and statistical analysis

Images taken by Zeiss Apotome 2 were processed with FIJI software. Contrast and image size of IF images were adjusted with Adobe photoshop. Expression profiles of genes of interest were produced in R. Bar graphs were generated by GraphPad Prism 7. All figures were made in Adobe Illustrator CS6. Sample size is mentioned in the excel sheets for the quantifications. For FACS analysis, for *Hes5::GFP*, only the bright GFP^+^ cells were collected. For *Tbr2::GFP*, both the bright and dim GFP^+^ cells were collected. For IF images, three fields of views were analyzed and quantified per sample. In IUE experiment analysis, the quantifications were also performed for GFP^+^ cells, to analyze the cell autonomous effects. Unpaired t-tests were used for most studies. The cut-off value for statistical significance are indicated in corresponding figure legend.

## Supplementary information

RNA-Seq and ISMARA data.

Supplementary information.
