# Supplementary material for: Tead transcription factors differentially regulate cortical development
Source: Sci Rep. 2020 Mar 13;10:4625. doi: 10.1038/s41598-020-61490-5 (PMC7070074; doi:10.1038/s41598-020-61490-5)
Supplement: Supplementary file 2 — Supplementary information. [file 41598_2020_61490_MOESM2_ESM.pdf]

Tead transcription factors differentially regulate cortical development

Tanzila Mukhtar[1], Jeremie Breda[2,3], Zahra Karimaddini[3,4], Pascal Grobecker[2,3], Alice Grison[1], Dagmar Iber[3,4], Christian Beisel[5], Erik van Nimwegen[2,3] and Verdon Taylor[1,*]

Affiliations:

[1] Department of Biomedicine, University of Basel, Mattenstrasse 28, CH-4058 Basel, Switzerland.

[2] Biozentrum, University of Basel, Klingelbergstrasse 50-70, CH-4056 Basel, Switzerland.

[3] Swiss Institute of Bioinformatics (SIB), Mattenstrasse 26, 4058 Basel, Switzerland.

[4] Computational Biology Group, D-BSSE, ETH Zürich, Mattenstrasse 26, CH-4058 Basel, Switzerland.

[5] Department of Biosystems Science and Engineering, ETH Zürich, 4058 Basel, Switzerland.

***Corresponding author:** Tel: +41 612075091; E-mail: verdon.taylor@unibas.ch

Supplementary Figures and Figure Legends

Figure S1

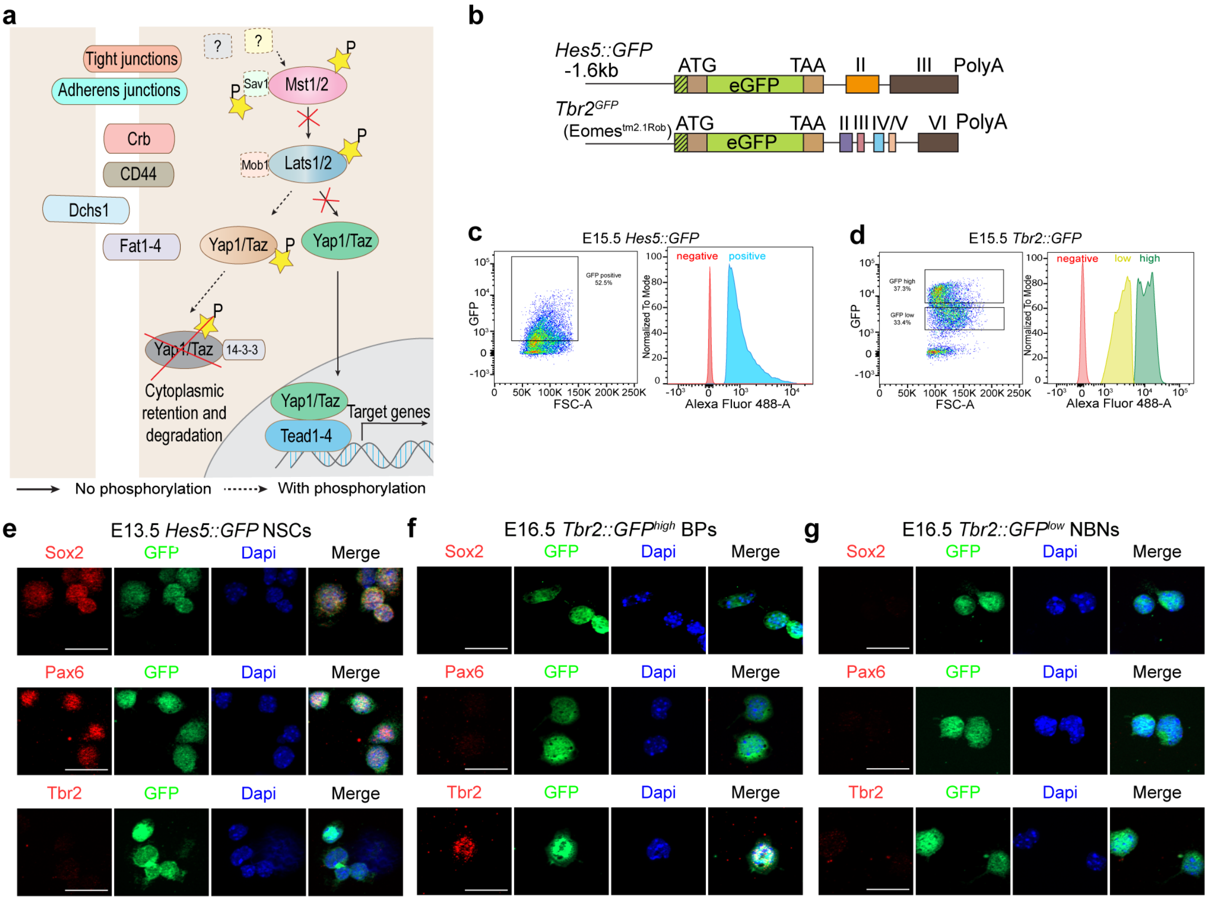

Figure S1: Validation for RNA sequencing paradigm and transgenic mice.

a. Schematic representation of Hippo signaling cascade. Illustration depicts series of sequential phosphorylation steps for co-activators Yap1/Taz, mediated by Mst1/2 and Lats1/2 kinases. If Hippo signaling is on, Yap1/Taz is phosphorylated, retained in the cytoplasm and degraded. If Hippo signaling is off, Yap1/Taz escapes phosphorylation and translocates to the nucleus, where it binds its partner TFs, e.g. Teads, to regulate transcription.

b. *Hes5::GFP* and *Tbr2::GFP* transgenic mice used for cell isolation.

c. Example of FACS plot for GFP^+ cell sorting from *Hes5::GFP* dorsal cortex at E15.5. Left plot shows the gating used to select GFP^+ cells. Right plot shows the GFP^- (red) and GFP^+ (blue) cell populations.

d. Example of FACS plot for GFP^+ cell sorting from *Tbr2::GFP* dorsal cortex at E15.5. Left plot shows the gates used to select the two populations of GFP^+ cells. Right plot shows the GFP^- (red), GFP^{low} (yellow) and GFP^{high} (green) cell populations.

e-g. Expression of progenitor markers (Sox2 and Pax6) and basal progenitor marker Tbr2 by *Hes5::GFP*$^+$ neural stem cells (NSCs) and *Tbr2::GFP*^{high} basal progenitors (BP) and *Tbr2::GFP*^{low} newborn neurons (NBNs) cells after FAC sorting. Scale bar = 20μm.

Figure S2

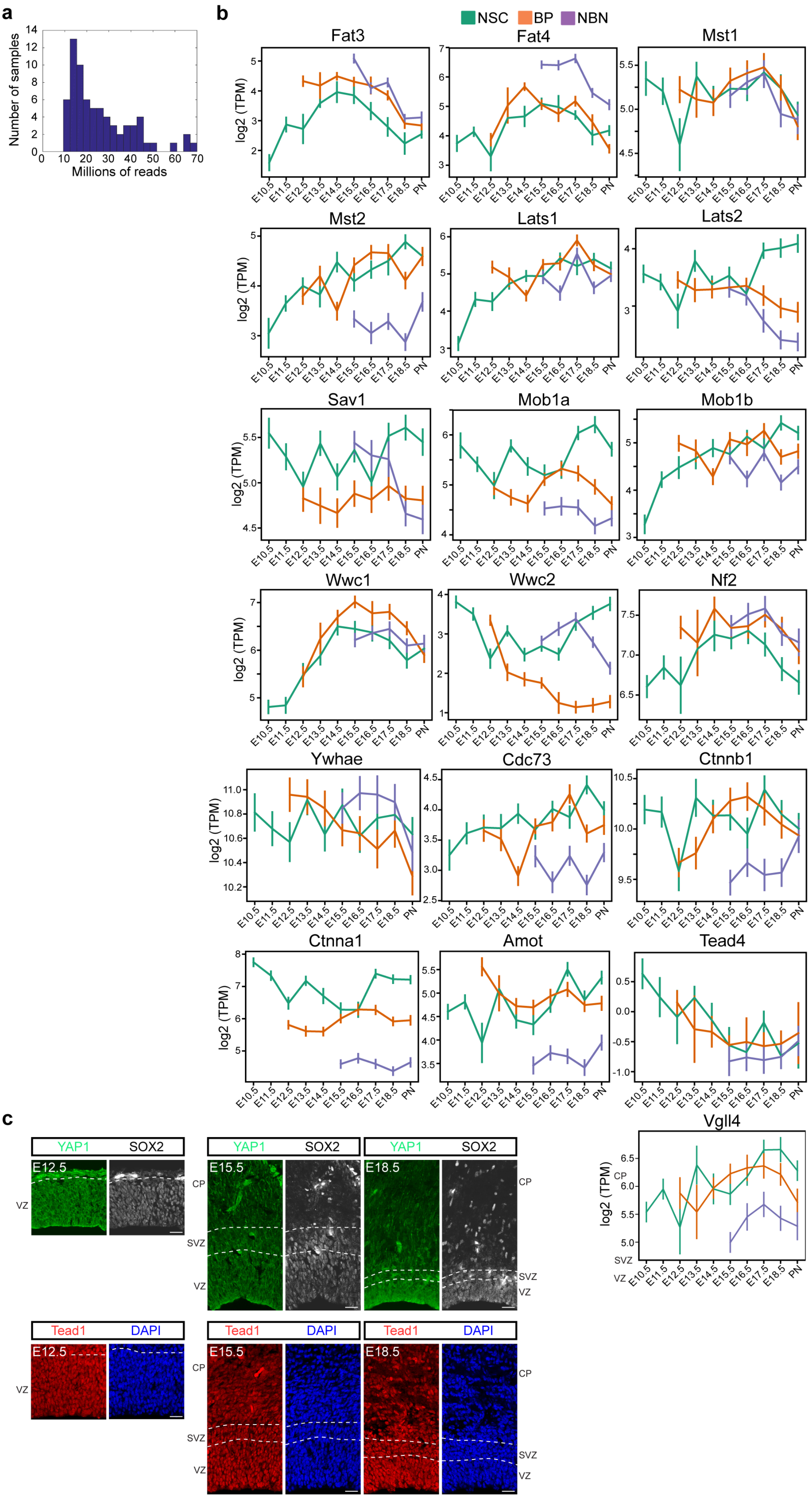
a
Number of samples
Millions of reads
b
NSC
BP
NBN
Fat3
Fat4
Mst1
Mst2
Lats1
Lats2
Sav1
Mob1a
Mob1b
Wwc1
Wwc2
Nf2
Ywhae
Cdc73
Ctnnb1
Ctnna1
Amot
Tead4
Vgll4
log2 (TPM)
E10.5
E11.5
E12.5
E13.5
E14.5
E15.5
E16.5
E17.5
E18.5
PN
c
YAP1
SOX2
Tead1
DAPI
E12.5
E15.5
E18.5
VZ
SVZ
CP

Figure S2: Transcriptional Dynamics of additional Hippo signaling effectors in NSCs, BPs and NBNs from RNA sequencing.

a. Graphical representation of number of total RNA-seq reads over the samples.

b. Expression profiles of components and intermediate effectors of Hippo signaling. Y-axis: mRNA level expressed as log2 TPM (transcripts per million).

c. Expression of Yap1 and Tead1 compared to the progenitor marker Sox2 in the developing cerebral cortex at E12.5, E15.5 and E18.5. Scale bars = 20μm.

Figure S3

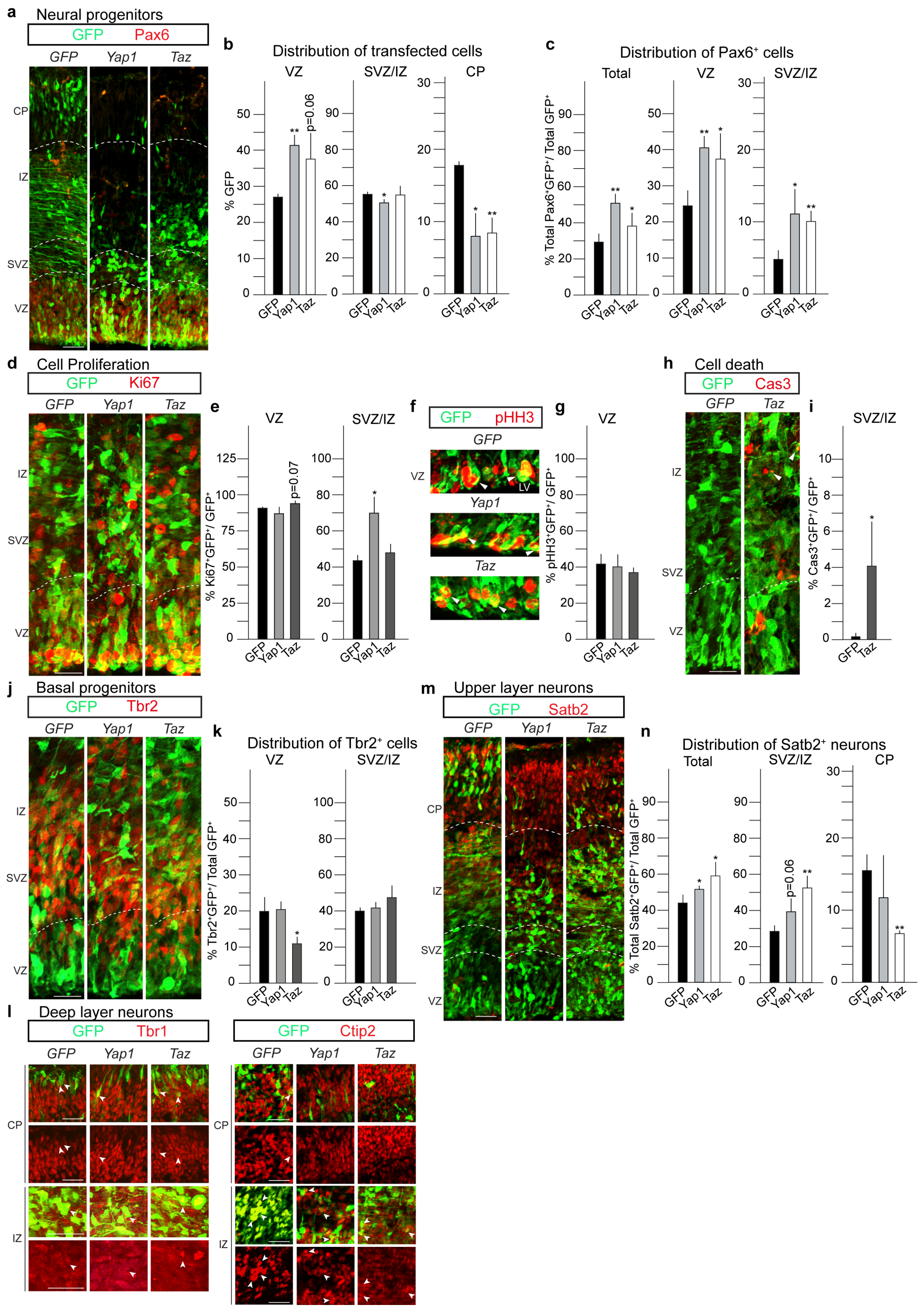

Figure S3: Co-activators Yap1 and Taz overexpression increase percentage of NSCs and reduce differentiation.

a. Coronal sections of transfected cortices immunostained for GFP and Pax6.

b. Quantification of distribution of GFP^+ transfected cells shows both Yap1 and Taz overexpression induce a similar phenotype.

c. Quantification of $Pax6^+GFP^+$ cells shows an increase in total $Pax6^+$ cells upon Yap1 and Taz overexpression, compared to GFP control in VZ, SVZ and total.

d. Coronal sections of transfected cortices immunostained for GFP and Ki67.

e. Quantification of $Ki67^+GFP^+$ cells shows no change in total $Ki67^+$ cells upon Yap1 and Taz overexpression, compared to GFP control in VZ, increase is observed in SVZ upon Yap1 overexpression.

f. Coronal sections of transfected cortices along the apical surface immunostained for GFP and pHH3.

g. Quantification of $pHH3^+GFP^+$ cells with their cell bodies lining the VZ shows no change in total $pHH3^+$ M-phase cells upon Yap1 and Taz overexpression, compared to GFP control in the apical VZ.

h. Coronal sections of transfected cortices immunostained for GFP and Cas3.

i. Quantification of $Cas3^+GFP^+$ cells shows increase in total $Cas3^+$ cells upon Taz overexpression, compared to GFP control in SVZ/IZ.

j. Coronal sections of transfected cortices immunostained for GFP and Tbr2.

k. Quantification of $Tbr2^+GFP^+$ cells shows a reduction in $Tbr2^+$ cells upon Taz overexpression compared to the GFP control.

l. Transfected cortices immunostained for GFP and Tbr1 or Ctip2, showing GFP^+ deep layer neurons marker positive cells in the CP and IZ.

m. Coronal sections of transfected cortices immunostained for GFP and Satb2, showing impaired migration of GFP^+ cells.

n. Quantification of distribution of GFP^+ transfected cells shows both Yap1 and Taz overexpression increase total $Satb2^+GFP^+$ cells in all cortical zones.

Summaries of the quantifications are in Table S1. Scale bars = 20 µm in d and j, 50 μm in a, h, l and m. Data are shown as average ± SEM, *p= 0.05, **p= 0.01.

Figure S4

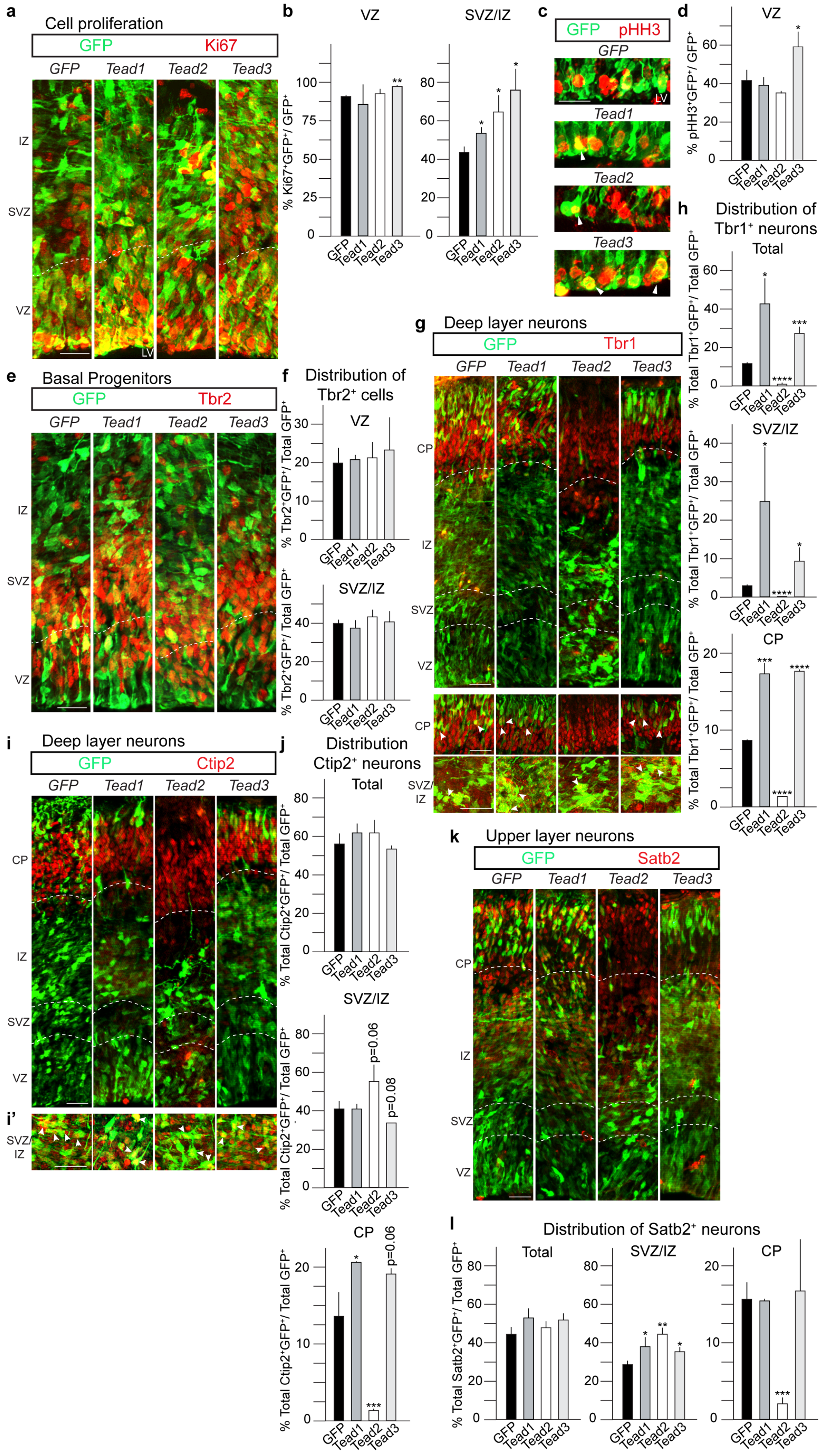

a
Cell proliferation
GFP Ki67
GFP Tead1 Tead2 Tead3
IZ
SVZ
VZ
LV
b
VZ
SVZ/IZ
% Ki67+GFP+/ GFP+
c
GFP pHH3
GFP
Tead1
Tead2
Tead3
d
VZ
% pHH3+GFP+/ GFP+
e
Basal Progenitors
GFP Tbr2
f
Distribution of Tbr2+ cells
% Tbr2+GFP+/ Total GFP+
g
Deep layer neurons
GFP Tbr1
CP
SVZ/IZ
h
Distribution of Tbr1+ neurons
Total
SVZ/IZ
CP
% Total Tbr1+GFP+/ Total GFP+
i
Deep layer neurons
GFP Ctip2
i'
j
Distribution Ctip2+ neurons
Total
SVZ/IZ
CP
p=0.06
p=0.08
% Total Ctip2+GFP+/ Total GFP+
k
Upper layer neurons
GFP Satb2
l
Distribution of Satb2+ neurons
Total
SVZ/IZ
CP
% Total Satb2+GFP+/ Total GFP+

Figure S4: Tead1, Tead2 and Tead3 overexpression affects cell fate and cortical layering.

a. Coronal sections of transfected cortices immunostained for GFP and Ki67.

b. Quantification of $Ki67^+GFP^+$ cells shows an increase in proliferation upon overexpression of Tead3 in VZ; and increase in proliferation in SVZ/IZ upon overexpression of all Tead1, Tead2 and Tead3.

c. Coronal sections of transfected cortices along the apical surface, immunostained for GFP and pHH3.

d. Quantification of $pHH3^+GFP^+$ cells with their cell bodies lining the VZ upon Tead1, Tead2 and Tead3 overexpression, compared to GFP control in the apical VZ.

e. Coronal sections of transfected cortices immunostained for GFP and Tbr2.

f. Quantification of $Tbr2^+GFP^+$ cells shows no change in BPs upon overexpression of Tead TFs.

g. Coronal sections of transfected cortices immunostained for GFP and Tbr1.

h. Quantification of distribution of GFP^+ transfected cells shows that both Tead1 and Tead3 overexpression induce a similar phenotype, an increase of $Tbr1^+GFP^+$ cells in SVZ/IZ, CP and total. Tead2 overexpression shows an opposite phenotype to Tead1 and Tead3, with less $Tbr1^+GFP^+$ cells in the CP and also a significant decrease in total.

i-i''. Coronal sections of transfected cortices immunostained for GFP and Ctip2.

j. Quantification of distribution of GFP^+ transfected cells shows both Tead1 and Tead3 overexpression induce a similar phenotype, an increase of $Ctip2^+GFP^+$ cells in CP and no changes in total.

k. Coronal sections of transfected cortices immunostained for GFP and Satb2.

l. Quantification of $Satb2^+GFP^+$ cells over total GFP^+ cells shows a decrease in $Satb2^+GFP^+$ cells in CP upon Tead2 overexpression, compared to GFP control. $Satb2^+GFP^+$ cells remain unchanged upon overexpression of Tead1 and Tead3.

Summaries of the quantifications are in Table S2. Scale bars = 50 μm. Data are shown as average $\pm$ SEM, *p= 0.05, **p= 0.01, ***p=0.001, ****<p=0.0001.

Figure S5

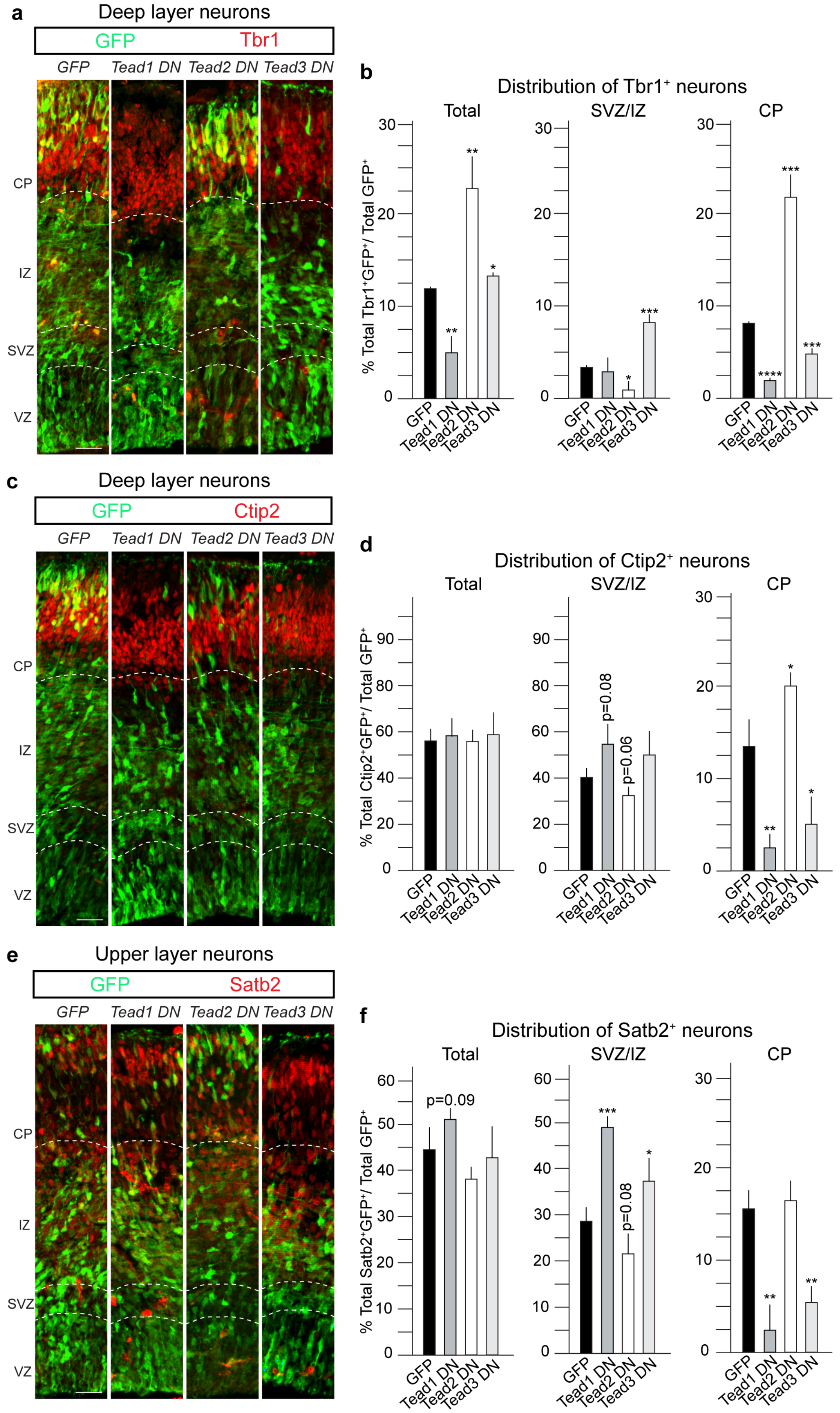

a
Deep layer neurons
GFP
Tbr1
GFP
Tead1 DN
Tead2 DN
Tead3 DN
CP
IZ
SVZ
VZ
b
Distribution of Tbr1⁺ neurons
Total
SVZ/IZ
CP
% Total Tbr1⁺GFP⁺/ Total GFP⁺
c
Deep layer neurons
GFP
Ctip2
d
Distribution of Ctip2⁺ neurons
% Total Ctip2⁺GFP⁺/ Total GFP⁺
p=0.08
p=0.06
e
Upper layer neurons
GFP
Satb2
f
Distribution of Satb2⁺ neurons
% Total Satb2⁺GFP⁺/ Total GFP⁺
p=0.09
p=0.08

Figure S5: Tead1 DN, Tead2 DN and Tead3 DN constructs affect cell fate and cortical layering.

a. Coronal sections of transfected cortices immunostained for GFP and Tbr1.

b. Quantification of fate changes in deep layer $Tbr1^{+}$ neurons, shows a decrease of $Tbr1^{+}GFP^{+}$ cells in CP upon Tead1 DN and Tead3 DN transfection. This is opposite to the phenotype with Tead2 DN, with an increase of $Tbr1^{+}GFP^{+}$ cells in CP. Similar changes are observed in total $Tbr1^{+}GFP^{+}$ cells.

c. Coronal sections of transfected cortices immunostained for GFP and Ctip2.

d. Quantification of fate changes in deep layer $Ctip2^{+}$ neurons, shows a decrease of $Ctip2^{+}GFP^{+}$ cells in CP upon Tead1 DN and Tead3 DN transfection. This is opposite to the phenotype with Tead2 DN, with an increase of $Ctip2^{+}GFP^{+}$ cells in CP. The total cell numbers remain unchanged; hence the manipulations only affect the migration of $Ctip2^{+}GFP^{+}$ cells.

e. Coronal sections of transfected cortices immunostained for GFP and Satb2.

f. Quantification of distribution of GFP^{+} transfected cells shows both Tead1 and Tead3 overexpression induce a similar phenotype, a decrease of $Satb2^{+}GFP^{+}$ cells in CP and no changes in total.

Summaries of the quantifications are in Table S3. Scale bars = 50 μm. Data are shown as average $\pm$ SEM, *p= 0.05, **p= 0.01, ***p=0.001, ****<p=0.0001.

Figure S6

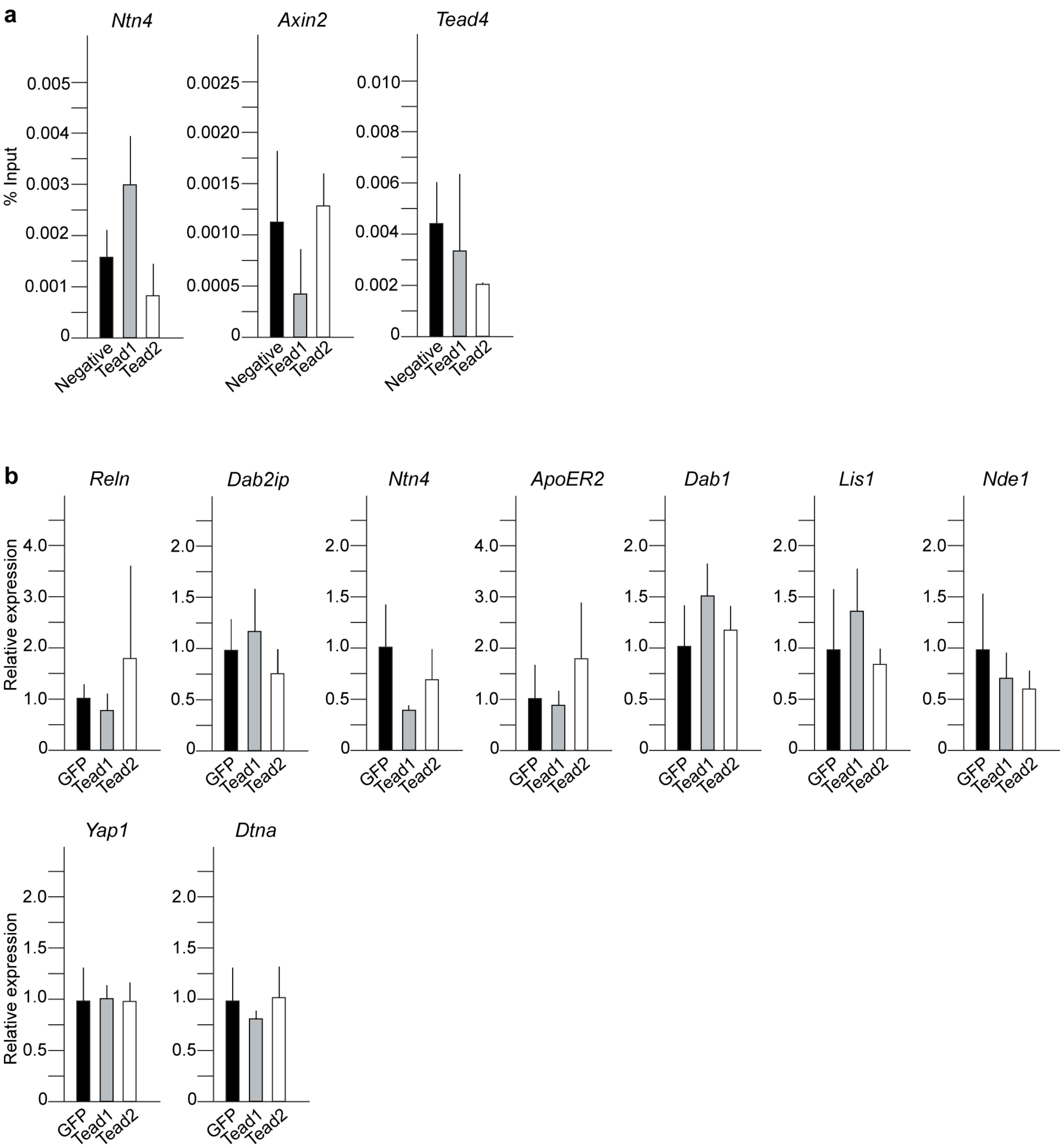

Figure S6: Additional ISMARA predicted targets tested by RT-qPCR and ChIP-PCR.

a. ChIP-qPCR does not pull-down Ntn4, Axin2 and Tead4 with both Tead1 and Tead2. An empty mCherry vector was used as the negative control.

b. Relative expression of Reln, Dab2ip, Ntn4, ApoER2, Dab1, Lis1, Nde1, Yap1 and Dtna show no significant changes upon overexpression of both Tead1 and Tead2.

Summaries of the quantifications are in Table S5. Data are shown as average ± SEM.

Figure S7

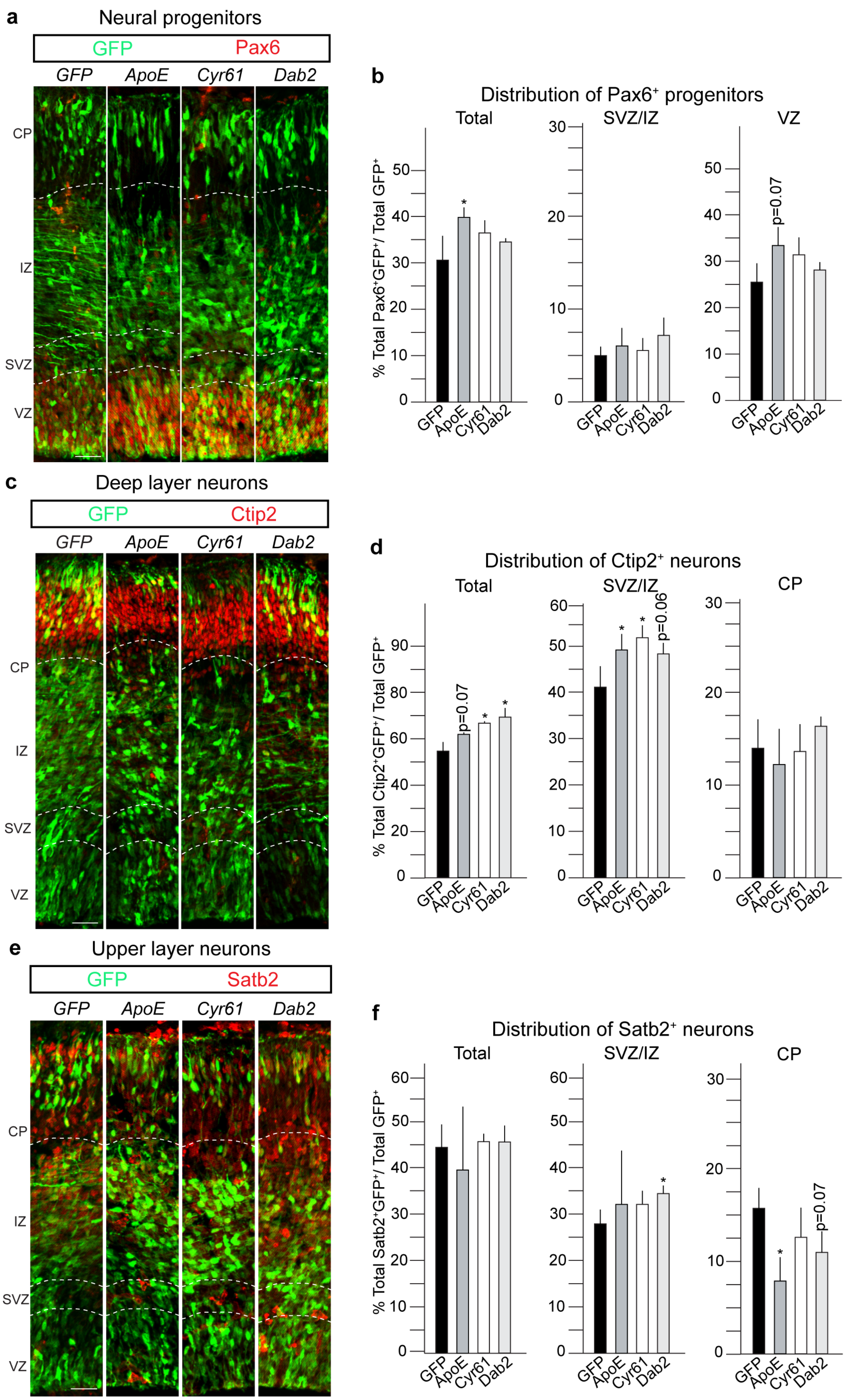
a
Neural progenitors
GFP
Pax6
GFP
ApoE
Cyr61
Dab2
CP
IZ
SVZ
VZ
b
Distribution of Pax6+ progenitors
Total
SVZ/IZ
VZ
% Total Pax6+GFP+/ Total GFP+
p=0.07
c
Deep layer neurons
GFP
Ctip2
d
Distribution of Ctip2+ neurons
Total
SVZ/IZ
CP
% Total Ctip2+GFP+/ Total GFP+
p=0.07
p=0.06
e
Upper layer neurons
GFP
Satb2
f
Distribution of Satb2+ neurons
Total
SVZ/IZ
CP
% Total Satb2+GFP+/ Total GFP+
p=0.07

Figure S7: Overexpression of ApoE, Cyr61 and Dab2 affects cell fate and cortical layering.

a. Coronal sections of transfected cortices immunostained for GFP and Pax6.

b. Quantification of $Pax6^{+}GFP^{+}$ cells shows an increase in total $Pax6^{+}$ cells upon ApoE overexpression.

c. Coronal sections of transfected cortices immunostained for GFP and Ctip2.

d. Quantification of fate changes in deep layer $Ctip2^{+}$ neurons, shows a slight increase of $Ctip2^{+}GFP^{+}$ cells in total upon Cyr61 and Dab2 overexpression.

e. Coronal sections of transfected cortices immunostained for GFP and Satb2.

f. Quantification of distribution of GFP^{+} transfected cells shows ApoE overexpression decreased $Satb2^{+}GFP^{+}$ cells in CP and no changes in total. Dab2 overexpression increases the $Satb2^{+}GFP^{+}$ cells in SVZ/IZ.

Summaries of the quantifications are in Table S6. Scale bars = 50 μm. Data are shown as average $\pm$ SEM, *p= 0.05.

Figure S8

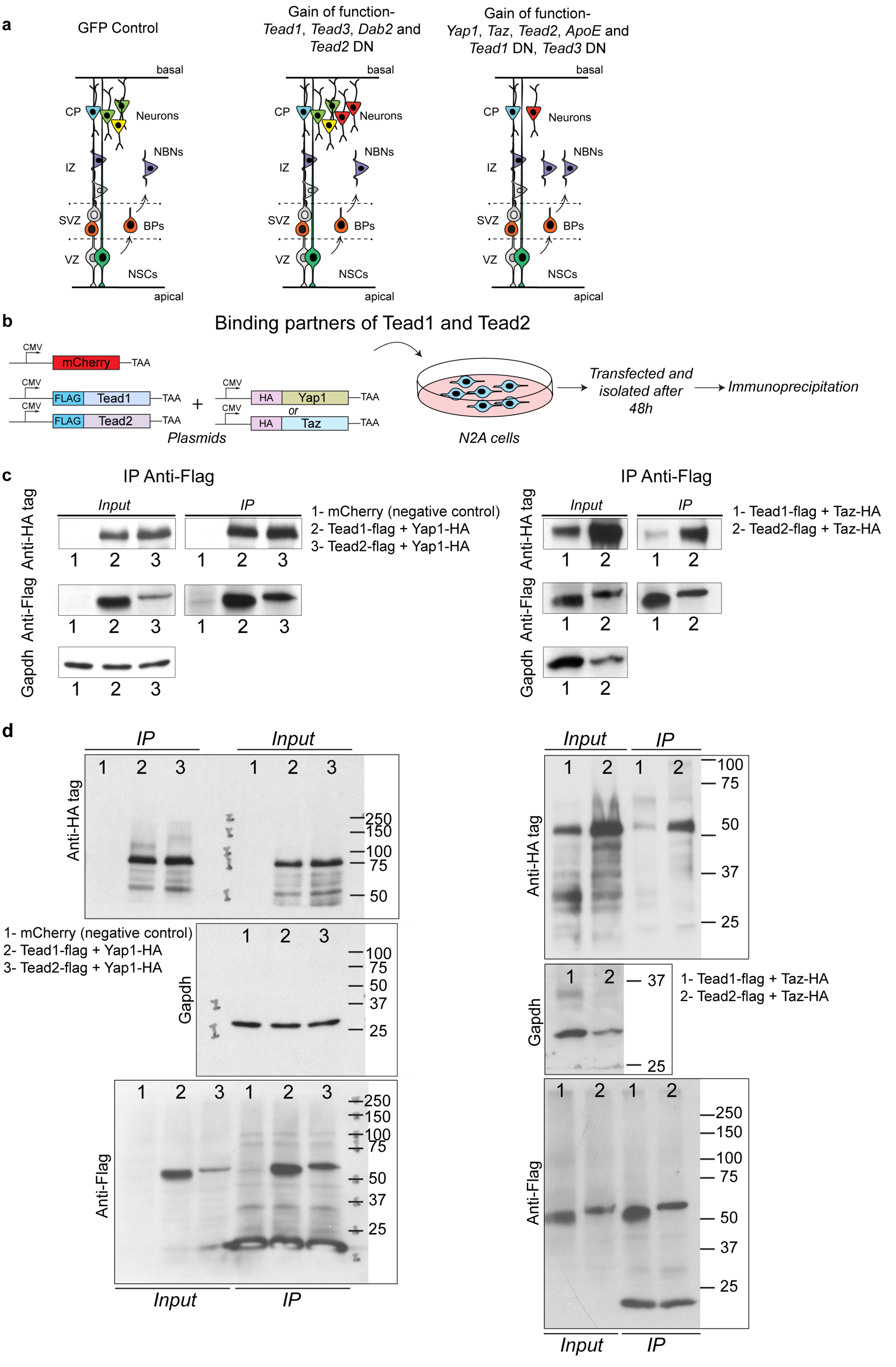
a
GFP Control
Gain of function-
Tead1, Tead3, Dab2 and Tead2 DN
Gain of function-
Yap1, Taz, Tead2, ApoE and Tead1 DN, Tead3 DN
basal
CP
Neurons
IZ
NBNs
SVZ
BPs
VZ
NSCs
apical
b
Binding partners of Tead1 and Tead2
CMV
mCherry
TAA
FLAG
Tead1
Tead2
HA
Yap1
or
Taz
Plasmids
N2A cells
Transfected and isolated after 48h
Immunoprecipitation
c
IP Anti-Flag
Input
IP
Anti-HA tag
Anti-Flag
Gapdh
1- mCherry (negative control)
2- Tead1-flag + Yap1-HA
3- Tead2-flag + Yap1-HA
1- Tead1-flag + Taz-HA
2- Tead2-flag + Taz-HA
d
250
150
100
75
50
37
25

Figure S8: Tead2 preferentially binds Yap1, in an overexpression paradigm.

a. Summary of the phenotypic effects upon gain and loss of function of Hippo effectors and targets on neuronal migration in the CP.

b. N2A cells were transfected with flag tagged-Tead1 or Tead2 expression constructs; co-transfected with HA-tagged Yap1 expression construct. Cells were lysed after 48 hours and immunoprecipitation was performed using Anti-Flag antibody.

c. Western blot detection revealed that Yap1 preferentially binds Tead2 in N2A cells. Cropped images of the complete blots shown in **d**.

d. Images of the full-length blots including, molecular weight markers of the, of the cropped blots shown in **c**.

Supplementary Data Tables

Table S1

a

	Average ± SEM (GFP^+ cells) E13.5-E15.5, 48 hour chase		
	VZ	SVZ/IZ	CP
GFP Control	28.11 ± 0.46	54.04 ± 1.00	17.85 ± 0.55
n	n=3	n=3	n=3
Yap1	52.45 ± 3.07	49.16 ± 1.41	8.35 ± 1.69
n	n=3	n=3	n=3
Taz	48.94 ± 4.65	53 ± 2.95	8.55 ± 1.31
n	n=3	n=3	n=3
P-Values			
Yap1 v's Control	0.0017 (**)	0.0466 (*)	0.0102 (*)
Taz v's Control	0.066 (*)	0.7571 (ns)	0.0049 (**)

b

	Average ± SEM (GFP^+Pax6^+ cells)/Total GFP+ E13.5-E15.5, 48 hour chase		
	VZ	SVZ/IZ	Total
GFP Control	25.71 ± 2.40	4.90 ± 0.65	30.61 ± 2.87
n	n=3	n=3	n=3
Yap1	41.22 ± 1.57	11.24 ± 2.05	52.45 ± 3.10
n	n=3	n=3	n=3
Taz	38.45 ± 4.19	10.48 ± 0.90	48.94 ± 1.31
n	n=3	n=3	n=3
P-Values			
Yap1 v's Control	0.0066 (**)	0.0299 (*)	0.0069 (**)
Taz v's Control	0.05 (*)	0.006 (**)	0.0283 (*)

c

	Average ± SEM ($Ki67^+GFP^+$ cells)/Total GFP^+ E13.5-E15.5, 48 hour chase	
	VZ	SVZ/IZ
GFP Control	92.71 ± 0.43	43.7 ± 2.16
n	n=3	n=3
Yap1	90.75 ± 3.73	52.8 ± 1.75
n	n=3	n=3
Taz	96.96 ± 1.206	47.59 ± 2.8
n	n=3	n=3
P-Values		
Yap1 v's Control	0.6293 (ns)	0.0308 (*)
Taz v's Control	0.06 (ns)	0.322 (ns)

d

	Average ± SEM ($pHH3^+GFP^+$cells)/Total GFP^+ E13.5-E15.5, 48 hour chase
	VZ
GFP Control	42.01 ± 3.131
n	n=3
Yap1	40.83 ± 3.816
n	n=3
Taz	37.69 ± 1.404
n	n=3
P-Values	
Yap1 v's Control	0.8231 (ns)
Taz v's Control	0.2761 (ns)

e

	Average ± SEM ($Cas3^+GFP^+$ cells)/Total GFP^+ E13.5-E15.5, 48 hour chase
	SVZ/IZ
GFP Control	0.18 ± 0.11
n	n=3
Taz	3.97± 1.37
n	n = 3
P-Values	
Taz v's Control	0.05 (*)

f

	Average ± SEM (GFP^+Tbr2^+ cells)/Total GFP+ in respective zones E13.5-E15.5, 48 hour chase	
	VZ	SVZ/IZ
GFP Control	20.52 ± 2.19	40.64 ± 1.02
n	n=3	n=3
Yap1	21.29 ± 1.16	42.46 ± 1.73
n	n=3	n=3
Taz	11.73 ± 1.01	48.39 ± 3.57
n	n=3	n=3
P-Values		
Yap1 v's Control	0.7714 (ns)	0.4165 (ns)
Taz v's Control	0.0219 (*)	0.1052 (ns)

g

	Average ± SEM (GFP^+Tbr1^+ cells)/Total GFP^+ E13.5-E15.5, 48 hour chase		
	SVZ/IZ	CP	Total
GFP Control	3.66 ± 0.08	8.68 ± 0.07	12.34 ± 0.09
n	n=3	n=3	n=3
Yap1	2.88 ± 1.48	5.20 ± 0.36	8.08 ± 1.70
n	n=3	n=3	n=3
Taz	3.44 ± 0.91	4.83 ± 0.70	8.28 ± 0.26
n	n=3	n=3	n=3
P-Values			
Yap1 v's Control	0.2402 (ns)	0.011 (**)	0.0838 (*)
Taz v's Control	0.7319 (ns)	0.0099 (**)	0.0002 (***)

h

	Average ± SEM (GFP^+Ctip2^+ cells)/Total GFP^+ E13.5-E15.5, 48 hour chase			
	VZ	SVZ/IZ	CP	Total
GFP Control	1.87 ± 1.06	41.39 ± 2.47	13.98 ± 1.82	57.24 ± 3.02
n	n=3	n=3	n=3	n=3
Yap1	7.58 ± 2.90	53.40 ± 3.2	6.46 ± 1.36	67.44 ± 4.23
n	n=3	n=3	n=3	n=3
Taz	6.96 ± 1.68	56.21 ± 5.42	5.06 ± 1.10	70.32 ± 5.27
n	n=3	n=3	n=3	n=3
P-Values				
Yap1 v's Control	0.1082 (ns)	0.041 (*)	0.0289 (*)	0.1206 (ns)
Taz v's Control	0.07 (ns)	0.05 (*)	0.0126 (*)	0.1046 (ns)

i

	Average ± SEM (GFP^+Satb2^+ cells)/Total GFP^+ E13.5-E15.5, 48 hour chase		
	SVZ/IZ	CP	Total
GFP Control	28.87 ± 1.81	15.89 ± 1.39	44.76 ± 2.78
n	n=3	n=3	n=3
Yap1	40.59 ± 4.45	12.00 ± 3.40	52.59 ± 1.05
n	n=3	n=3	n=3
Taz	53.15 ± 3.89	6.90 ± 0.31	60.05 ± 4.20
n	n=3	n=3	n=3
P-Values			
Yap1 v's Control	0.06 (ns)	0.3423 (ns)	0.05 (*)
Taz v's Control	0.0047 (**)	0.0021 (**)	0.04 (*)

Table S1: Quantification of phenotypic changes upon overexpression of co-activators Yap1 and Taz (refers to Fig. 2).

a. Table showing the distribution of transfected cells upon overexpression of Yap1 and Taz, compared to GFP control. **b.** Table showing quantification for $Pax6^+GFP^+$ cells upon overexpression of Yap1 and Taz, compared to GFP control **c.** Table showing quantification for $Ki67^+GFP^+$ cells upon overexpression of Yap1 and Taz, compared to GFP control. **d.** Table showing quantification for $pHH3^+GFP^+$ cells upon overexpression of Yap1 and Taz, compared to GFP control. **e.** Table showing quantification for $Cas3^+GFP^+$ cells upon overexpression of Yap1 and Taz, compared to GFP control. **f.** Table showing quantification for $Tbr2^+GFP^+$ cells upon overexpression of Yap1 and Taz, compared to GFP control. **g.** Table showing quantification for $Tbr1^+GFP^+$ cells upon overexpression of Yap1 and Taz, compared to GFP control. **h.** Table showing quantification for $Ctip2^+GFP^+$ cells upon overexpression of Yap1 and Taz, compared to GFP control. **i.** Table showing quantification for $Satb2^+GFP^+$ cells upon overexpression of Yap1 and Taz, compared to GFP control.

Table S2

a

	**Average ± SEM (GFP^+ cells) E13.5-E15.5, 48 hour chase**		
	VZ	SVZ/IZ	CP
GFP Control	28.11 ± 0.46	54.04 ± 0.99	17.85 ± 0.55
n	n=3	n=3	n=3
Tead1	33.38 ± 1.87	43.99 ± 1.57	24.44 ± 1.33
n	n=3	n=3	n=3
Tead2	35.34 ± 1.54	64.08 ± 1.70	0.59 ± 0.22
n	n=3	n=3	n=3
Tead3	37.43 ± 1.62	36.33 ± 1.89	26.25 ± 0.75
n	n=3	n=3	n=3
P-Values			
Tead1 v's Control	0.05 (*)	0.0057 (**)	0.009 (***)
Tead2 v's Control	0.3129 (ns)	0.0072 (**)	<0.0001 (****)
Tead3 v's Control	0.0048 (**)	0.0012 (**)	0.0008 (***)

b

	**Average ± SEM (GFP^+Pax6^+ cells)/Total GFP^+ E13.5-E15.5, 48 hour chase**		
	VZ	SVZ/IZ	Total
GFP Control	25.71 ± 2.40	4.90 ± 0.65	30.61 ± 2.87
n	n=3	n=3	n=3
Tead1	31.74 ± 1.21	3.20 ± 0.63	34.95 ± 1.48
n	n=3	n=3	n=3
Tead2	32.21 ± 3.49	7.06 ± 1.98	39.26 ± 5.46
n	n=3	n=3	n=3
Tead3	37.43 ± 1.62	3.73 ± 0.57	41.15 ± 1.99
n	n=3	n=3	n=3
P-Values			
Tead1 v's Control	0.0927 (ns)	0.1396 (ns)	0.2512 (ns)
Tead2 v's Control	0.2016 (ns)	0.4088 (ns)	0.2361 (ns)
Tead3 v's Control	0.0173 (*)	0.2385 (ns)	0.0412 (*)

c

	**Average ± SEM (GFP^+Ki67^+ cells)/Total GFP^+ E13.5-E15.5, 48 hour chase**	
	VZ	SVZ/IZ
GFP Control	92.71 ± 0.43	43.7 ± 2.16
n	n=3	n=3
Tead1	88.99 ± 5.17	52.8 ± 1.75
n	n=3	n=3
Tead2	94.94 ± 1.95	65.29 ± 4.89
n	n=3	n=3
Tead3	98.49 ± 1.27	75.48 ± 5.78
n	n=3	n=3
P-Values		
Tead1 v's Control	0.5135 (ns)	0.0308 (*)
Tead2 v's Control	0.3271 (ns)	0.0156 (*)
Tead3 v's Control	0.0126 (*)	0.0068 (**)

d

	Average ± SEM (GFP^+pHH3^+ cells)/Total GFP^+ E13.5-E15.5, 48 hour chase
	VZ
GFP Control	42.01 ± 3.131
n	n=3
Tead1	39.87 ± 2.112
n	n=3
Tead2	36.02 ± 0.5645
n	n=3
Tead3	60.03 ± 4.433
n	n=3
P-Values	
Tead1 v's Control	0.6015 (ns)
Tead2 v's Control	0.133 (ns)
Tead3 v's Control	0.0294 (*)

e

	Average ± SEM (GFP^+Tbr2^+ cells)/Total GFP^+ in respective zones E13.5-E15.5, 48 hour chase	
	VZ	SVZ/IZ
GFP Control	20.52 ± 2.188	40.64 ± 1.025
n	n=3	n=3
Tead1	21.47 ± 0.6866	38.61 ± 2.317
n	n=3	n=3
Tead2	22.13 ± 2.39	44.46 ± 2.01
n	n=3	n=3
Tead3	24.61 ± 4.768	42.03 ± 3.251
n	n=3	n=3
P-Values		
Tead1 v's Control	0.6983 (ns)	0.4678 (ns)
Tead2 v's Control	0.6445 (ns)	0.166 (ns)
Tead3 v's Control	0.4784 (ns)	0.7043 (ns)

f

	Average ± SEM (GFP^+Tbr1^+ cells)/Total GFP^+ E13.5-E15.5, 48 hour chase		
	VZ	SVZ/IZ	Total
GFP Control	3.66 ± 0.08	8.68 ± 0.07	12.34 ± 0.09
n	n=3	n=3	n=3
Tead1	25.71 ± 8.39	17.53 ± 0.88	43.24 ± 8.36
n	n=3	n=3	n=3
Tead2	0.00 ± 0.00	1.04 ± 0.52	1.57 ± 0.02
n	n=3	n=3	n=3
Tead3	10.22 ± 1.96	17.93 ± 0.20	28.14 ± 2.10
n	n=3	n=3	n=3
P-Values			
Tead1 v's Control	0.0382 (*)	0.0003 (***)	0.014 (*)
Tead2 v's Control	<0.0001 (****)	<0.0001 (****)	<0.0001 (****)
Tead3 v's Control	0.0145 (*)	<0.0001 (****)	0.001 (***)

g

Average ± SEM (GFP⁺Ctip2⁺ cells)/Total GFP⁺
E13.5-E15.5, 48 hour chase

	VZ	SVZ/IZ	CP	Total
GFP Control	1.87 ± 1.06	41.39 ± 2.47	13.98 ± 1.82	57.24 ± 3.02
n	n=3	n=3	n=3	n=3
Tead1	1.18 ± 1.18	27.91 ± 13.99	20.96 ± 0.06	64.59 ± 3.4
n	n=3	n=3	n=3	n=3
Tead2	6.44 ± 1.42	56.21 ± 5.42	1.758 ± 0.26	64.40 ± 4.23
n	n=3	n=3	n=3	n=3
Tead3	0.44 ± 0.44	35.33 ± 0.95	18.98 ± 0.47	54.75 ± 1.03
n	n=3	n=3	n=3	n=3
P-Values				
Tead1 v's Control	0.6279 (ns)	0.7329 (ns)	0.0255 (*)	0.08 (ns)
Tead2 v's Control	0.07 (ns)	0.06 (ns)	0.001 (***)	0.2402 (ns)
Tead3 v's Control	0.3258 (ns)	0.08 (ns)	0.06 (ns)	0.479 (ns)

h

Average ± SEM (GFP⁺Satb2⁺ cells)/Total GFP⁺
E13.5-E15.5, 48 hour chase

	SVZ/IZ	CP	Total
GFP Control	28.87 ± 1.81	15.89 ± 1.39	44.76 ± 2.78
n	n=3	n=3	n=3
Tead1	37.24 ± 2.51	15.44 ± 0.18	52.68 ± 2.68
n	n=3	n=3	n=3
Tead2	45.79 ± 2.31	2.19 ± 0.56	47.97 ± 2.24
n	n=3	n=3	n=3
Tead3	35.53 ± 1.50	16.9 ± 3.86	52.43 ± 2.40
n	n=3	n=3	n=3
P-Values			
Tead1 v's Control	0.05 (*)	0.7957 (ns)	0.1097 (ns)
Tead2 v's Control	0.0045 (**)	0.0008 (***)	0.4181 (ns)
Tead3 v's Control	0.047 (*)	0.8589 (ns)	0.1051 (ns)

Table S2: Quantification of phenotypic changes upon overexpression of TFs Tead1, Tead2 and Tead3 (refers to Fig. 3).

a. Table showing the distribution of transfected cells upon overexpression of Tead1, Tead2 and Tead3 compared to GFP control. **b.** Table showing quantification for $Pax6^+GFP^+$ cells upon overexpression of Tead1, Tead2 and Tead3, compared to GFP control. **c.** Table showing quantification for $Ki67^+GFP^+$ cells upon overexpression of Tead1, Tead2 and Tead3, compared to GFP control. **d.** Table showing quantification for $pHH3^+GFP^+$ cells upon overexpression of Tead1, Tead2 and Tead3, compared to GFP control. **e.** Table showing quantification for $Tbr2^+GFP^+$ cells upon overexpression of Tead1, Tead2 and Tead3, compared to GFP control. **f.** Table showing quantification for $Tbr1^+GFP^+$ cells upon overexpression of Tead1, Tead2 and Tead3, compared to GFP control. **g.** Table showing quantification for $Ctip2^+GFP^+$ cells upon overexpression of Tead1, Tead2 and Tead3, compared to GFP control **h.** Table showing quantification for $Satb2^+GFP^+$ cells upon overexpression of Tead1, Tead2 and Tead3, compared to GFP control.

Table S3

a

	**Average ± SEM (GFP$^+$ cells) E13.5-E15.5, 48 hour chase**		
	VZ	SVZ/IZ	CP
GFP Control	28.11 ± 0.46	54.04 ± 0.99	17.85 ± 0.55
n	n=3	n=3	n=3
Tead1 DN	38.59 ± 1.29	57.53 ± 1.93	3.89 ± 0.88
n	n=3	n=3	n=3
Tead2 DN	32.36 ± 0.90	38.46 ± 1.07	29.19 ± 1.68
n	n=3	n=3	n=3
Tead3 DN	36.59 ± 3.14	54.58 ± 3.10	8.832 ± 1.07
n	n=3	n=3	n=3
P-Values			
Tead1 DN v's Control	0.0014 (**)	0.1833 (ns)	0.0006 (***)
Tead2 DN v's Control	0.013 (*)	0.0004 (***)	0.0024 (**)
Tead3 DN v's Control	0.05 (*)	0.8746 (ns)	0.0028 (**)

b

	**Average ± SEM (GFP$^+$Pax6$^+$ cells)/Total GFP$^+$ E13.5-E15.5, 48 hour chase**		
	VZ	SVZ/IZ	Total
GFP Control	25.71 ± 2.40	4.90 ± 0.65	30.61 ± 2.87
n	n=3	n=3	n=3
Tead1 DN	32.36 ± 0.90	6.30 ± 0.79	39.29 ± 1.23
n	n=3	n=3	n=3
Tead2 DN	38.58 ± 1.29	8.42 ± 1.05	47.00 ± 2.08
n	n=3	n=3	n=3
Tead3 DN	36.59 ± 3.14	5.06 ± 0.56	41.65 ± 3.10
n	n=3	n=3	n=3
P-Values			
Tead1 DN v's Control	0.06 (*)	0.1637 (ns)	0.05 (*)
Tead2 DN v's Control	0.0107 (*)	0.0413 (*)	0.0108 (*)
Tead3 DN v's Control	0.05 (*)	0.8484 (ns)	0.06 (*)

c

	**Average ± SEM (GFP$^+$Tbr1$^+$ cells)/Total GFP$^+$ E13.5-E15.5, 48 hour chase**		
	SVZ/IZ+	CP+	Total+
GFP Control	3.66 ± 0.08	8.68 ± 0.06	12.34 ± 0.09
n	n=3	n=3	n=3
Tead1 DN	3.21 ± 1.01	2.22 ± 0.19	5.42 ± 1.02
n	n=3	n=3	n=3
Tead2 DN	1.04 ± 0.63	22.44 ± 1.40	23.48 ± 2.00
n	n=3	n=3	n=3
Tead3 DN	8.53 ± 0.64	5.20 ± 0.27	13.73 ± 0.38
n	n=3	n=3	n=3
P-Values			
Tead1 DN v's Control	0.6025 (ns)	<0.0001 (****)	0.006 (**)
Tead2 DN v's Control	0.041 (*)	0.0003 (***)	0.0036 (**)
Tead3 DN v's Control	0.0009 (***)	0.0004 (***)	0.224 (ns)

d

	Average ± SEM (GFP^+Ctip2^+ cells)/Total GFP^+ E13.5-E15.5, 48 hour chase			
	VZ	SVZ/IZ	CP	Total
GFP Control	1.87 ± 1.06	41.39 ± 2.47	13.98 ± 1.82	57.24 ± 3.02
n	n=3	n=3	n=3	n=3
Tead1 DN	3.45 ± 0.69	54.43 ± 5.15	2.57 ± 0.89	59.59 ± 4.99
n	n=3	n=3	n=3	n=3
Tead2 DN	1.00 ± 0.51	33.09 ± 2.25	20.74 ± 0.85	57.30 ± 2.66
n	n=3	n=3	n=3	n=3
Tead3 DN	2.84 ± 1.03	50.45 ± 6.00	5.22 ± 1.68	60.02 ± 5.14
n	n=3	n=3	n=3	n=3
P-Values				
Tead1 DN v's Control	0.2817 (ns)	0.08 (ns)	0.0054 (**)	0.7061 (ns)
Tead2 DN v's Control	0.6334 (ns)	0.06 (ns)	0.035 (*)	0.9913 (ns)
Tead3 DN v's Control	0.5067 (ns)	0.2355 (ns)	0.0285 (*)	0.6585 (ns)

e

	Average ± SEM (GFP^+Satb2^+ cells)/Total GFP^+ E13.5-E15.5, 48 hour chase		
	SVZ/IZ	CP	Total
GFP Control	28.87 ± 1.81	15.89 ± 1.39	44.76 ± 2.78
n	n=3	n=3	n=3
Tead1 DN	49.14 ± 1.31	2.51 ± 1.56	51.65 ± 1.52
n	n=3	n=3	n=3
Tead2 DN	21.99 ± 2.32	16.75 ± 1.25	38.74 ± 1.55
n	n=3	n=3	n=3
Tead3 DN	37.68 ± 2.87	5.55 ± 1.04	43.24 ± 3.90
n	n=3	n=3	n=3
P-Values			
Tead1 DN v's Control	0.0066 (**)	0.0299 (*)	0.095 (ns)
Tead2 DN v's Control	0.08 (ns)	0.6678 (ns)	0.1312 (ns)
Tead3 DN v's Control	0.05 (*)	0.04 (**)	0.7638 (ns)

Table S3: Quantification of phenotypic changes upon expression of dominant negative forms of TFs Tead1, Tead2 and Tead3 (refers to Fig. 4).

a. Table showing the distribution of transfected cells upon expression of dominant negative forms of Tead1, Tead2 and Tead3, compared to GFP control. **b.** Table showing quantification for $Pax6^+GFP^+$ cells upon expression of dominant negative forms of Tead1, Tead2 and Tead3, compared to GFP control. **c.** Table showing quantification for $Tbr1^+GFP^+$ cells upon expression of dominant negative forms of Tead1, Tead2 and Tead3, compared to GFP control. **d.** Table showing quantification for $Ctip2^+GFP^+$ cells upon expression of dominant negative forms of Tead1, Tead2 and Tead3, compared to GFP control. **e.** Table showing quantification for $Satb2^+GFP^+$ cells upon expression of dominant negative forms of Tead1, Tead2 and Tead3, compared to GFP control.

Table S4

a

	Average ± SEM (GFP$^+$ cells) E13.5-E15.5, 48 hour chase		
	VZ	SVZ/IZ	CP
GFP Control	28.11 ± 0.46	54.04 ± 0.99	17.85 ± 0.55
n	n=3	n=3	n=3
Tead1 VP16	46.8 ± 4.55	45.36 ± 4.66	7.72 ± 0.08
n	n=3	n=3	n=3
Tead2 VP16	48.34 ± 17.68	33.63 ± 13.64	1.89 ± 0.61
n	n=3	n=3	n=3
P-Values			
Tead1 VP16 v's Control	0.0136 (*)	0.1426 (*)	<0.0001 (****)
Tead2 VP16 v's Control	0.32 (ns)	0.2091 (ns)	0.0002 (***)

b

	Average ± SEM (GFP$^+$Pax6$^+$ cells)/Total GFP$^+$ E13.5-E15.5, 48 hour chase		
	VZ	SVZ/IZ	Total
GFP Control	25.71 ± 2.40	4.90 ± 0.65	30.61 ± 2.87
n	n=3	n=3	n=3
Tead1 VP16	46.92 ± 4.59	4.19 ± 1.03	51.12 ± 3.60
n	n=3	n=3	n=3
Tead2 VP16	48.22 ± 17.76	29.13 ± 14.14	77.35 ± 15.58
n	n=3	n=3	n=3
P-Values			
Tead1 VP16 v's Control	0.0144 (*)	0.5473 (ns)	0.0115 (*)
Tead2 VP16 v's Control	0.278 (ns)	0.1153 (ns)	0.0476 (*)

Table S4: Quantification of phenotypic changes upon expression of transactive forms of TFs Tead1 and Tead2 (refers to Fig. 5).

a. Table showing the distribution of transfected cells upon expression of transactive forms of Tead1 and Tead2, compared to GFP control. **b.** Table showing quantification for Pax6$^+$GFP$^+$ cells upon expression of transactive forms of Tead1 and Tead2, compared to GFP control.

Table S5

a

**Chromatin Immunoprecipitation**	**Negative**	**Tead1-flag**	**Tead2-flag**
ApoE	0.0002373 ± 0	0.002739 ± 0.00019	0.004509 ± 0.00029
P-Values (t-test)		0.0063	0.0048
n	n=2	n=2	n=2
Dab2	0.0007925 ± 4.35e-8	0.00274 ± 4.4e-7	0.00335 ± 0.001357
P-Values (t-test)		<0.0001	0.2002
n	n=2	n=2	n=2
Cyr61	0.001553 ± 2.55e-8	0.003421 ± 2.65e-7	0.00335 ± 0.001357
P-Values (t-test)		<0.0001	0.08
n	n=2	n=2	n=2

b

Gene		Relative expression ± SEM (NSCs)	
	GFP Control	Tead1	Tead2
ApoE	1.11 ± 0.11	16.67 ± 0.70	31.43 ± 3.31
P-Values (t-test)		<0.0001	<0.0001
n	n=4	n=3	n=4
Dab2	0.65 ± 0.16	26.80 ± 9.85	38.95 ± 4.80
P-Values (t-test)		0.0378	0.0002
n	n=4	n=3	n=4
Cyr61	0.989 ± 0.12	5.99 ± 1.11	5.66 ± 1.14
P-Values (t-test)		0.0001	<0.0001
n	n=4	n=3	n=4
Dtna	1 ± 0.18	0.81 ± 0.03	1.01 ± 0.17
P-Values (t-test)		0.2332	0.9455
n	n=3	n=5	n=3
Reln	1 ± 0.14	0.80 ± 0.13	1.8 ± 0.9
P-Values (t-test)		0.3777	0.4939
n	n = 3	n = 5	n = 4
Dab2ip	1 ± 0.14	1.17 ± 0.15	0.77 ± 0.10
P-Values (t-test)		0.4339	0.2133
n	n = 5	n = 7	n = 5
Ntn4	1 ± 0.23	0.38 ± 0.024	0.666 ± 0.16
P-Values (t-test)		0.128	0.2978
n	n=3	n=2	n=3
ApoER2	1 ± 0.35	0.88 ± 0.10	1.76 ± 0.52
P-Values (t-test)		0.7007	0.3163
n	n=3	n=5	n=4
Dab1	1 ± 0.22	1.51 ± 0.14	1.18 ± 0.11
P-Values (t-test)		0.0821	0.4571
n	n=3	n=5	n=4
Lis1	1 ± 0.35	1.41 ± 0.18	0.87 ± 0.07
P-Values (t-test)		0.2891	0.681
n	n=3	n=5	n=4
Nde1	1 ± 0.33	0.74 ± 0.10	0.62 ± 0.08
P-Values (t-test)		0.3788	0.249
n	n=3	n=5	n=4
Yap1	1 ± 0.19	1.043 ± 0.06	0.99 ± 0.082
P-Values (t-test)		0.7952	0.9787
n	n=3	n=5	n=4

Table S5: Quantification of ChIP and relative expression for predicted Tead target genes (refers to Fig. 6).

a. Table summarizes ChIP results for flag tagged-Tead1 and flag tagged-Tead2 compared to negative control. **b.** Table summarizes relative expression of predicted target genes upon overexpression of Tead1 and Tead2 *in vivo*, compared to a GFP control.

Table S6

a

	Average ± SEM (GFP^+ cells) E13.5-E15.5, 48 hour chase		
	VZ	SVZ/IZ	CP
GFP Control	28.11 ± 0.46	54.04 ± 0.99	17.85 ± 0.55
n	n=3	n=3	n=3
ApoE	33.85 ± 2.37	53.83 ± 1.40	12.32 ± 0.99
n	n=3	n=3	n=3
Dab2	28.35 ± 0.88	51.16 ± 0.91	20.49 ± 0.42
n	n=3	n=3	n=3
Cyr61	31.44 ± 2.19	50.08 ± 0.33	16.69 ± 0.73
n	n=3	n=3	n=3
P-Values			
ApoE v's Control	0.07 (ns)	0.0906 (ns)	0.0101 (*)
Dab2 v's Control	0.82 (ns)	0.0986 (ns)	0.0194 (*)
Cyr61 v's Control	0.214 (ns)	0.0189 (*)	0.273 (ns)

b

	Average ± SEM (GFP^+Pax6^+ cells)/Total GFP^+ E13.5-E15.5, 48 hour chase		
	VZ	SVZ/IZ	Total
GFP Control	25.71 ± 2.40	4.90 ± 0.65	30.61 ± 2.87
n	n=3	n=3	n=3
ApoE	33.85 ± 2.37	5.99 ± 1.19	39.84 ± 1.18
n	n=3	n=3	n=3
Dab2	28.35 ± 0.88	7.04 ± 1.19	35.39 ± 0.60
n	n=3	n=3	n=3
Cyr61	31.44 ± 2.19	5.55 ± 0.79	36.99 ± 1.45
n	n=3	n=3	n=3
P-Values			
ApoE v's Control	0.07 (ns)	0.5027 (ns)	0.0441 (*)
Dab2 v's Control	0.3585 (ns)	0.195 (ns)	0.1817 (ns)
Cyr61 v's Control	0.1563 (ns)	0.5511 (ns)	0.1216 (ns)

c

	Average ± SEM (GFP^+Tbr1^+ cells)/Total GFP^+ E13.5-E15.5, 48 hour chase		
	SVZ/IZ	CP	Total
GFP Control	3.66 ± 0.08	8.67 ± 0.07	12.34 ± 0.09
n	n=3	n=3	n=3
ApoE	5.49 ± 0.80	10.53 ± 2.33	16.02 ± 3.00
n	n=3	n=3	n=3
Dab2	6.18 ± 1.13	16.66 ± 1.35	22.83 ± 2.01
n	n=3	n=3	n=3
Cyr61	6.00 ± 0.96	15.91 ± 0.14	21.9 ± 0.91
n	n=3	n=3	n=3
P-Values			
ApoE v's Control	0.08 (ns)	0.4972 (ns)	0.2927 (ns)
Dab2 v's Control	0.08 (ns)	0.0026 (**)	0.0043 (**)
Cyr61 v's Control	0.06 (ns)	<0.0001 (****)	0.0003 (***)

d

	Average ± SEM (GFP^+Ctip2^+ cells)/Total GFP^+ E13.5-E15.5, 48 hour chase			
	VZ	SVZ/IZ	CP	Total
GFP Control	1.87 ± 1.06	41.39 ± 2.47	13.98 ± 1.82	57.24 ± 3.02
n	n=3	n=3	n=3	n=3
ApoE	2.74 ± 0.37	50.29 ± 1.58	11.28 ± 1.83	64.31 ± 0.12
n	n=3	n=3	n=3	n=3
Dab2	6.01 ± 1.51	48.51 ± 1.35	16.49 ± 0.55	71.01 ± 2.58
n	n=3	n=3	n=3	n=3
Cyr61	2.78 ± 0.77	52.22 ± 1.66	13.66 ± 1.67	68.66 ± 0.37
n	n=3	n=3	n=3	n=3
P-Values				
ApoE v's Control	0.5394 (ns)	0.03 (*)	0.03 (*)	0.078 (ns)
Dab2 v's Control	0.09 (ns)	0.06 (ns)	0.2646 (ns)	0.0262 (*)
Cyr61 v's Control	0.4552 (ns)	0.022 (*)	0.9106 (ns)	0.0189 (*)

e

	Average ± SEM (GFP^+Satb2^+ cells)/Total GFP^+ E13.5-E15.5, 48 hour chase		
	SVZ/IZ	CP	Total
GFP Control	28.87 ± 1.81	15.89 ± 1.39	44.76 ± 2.78
n	n=3	n=3	n=3
ApoE	32.79 ± 6.51	7.90 ± 1.58	40.68 ± 7.70
n	n=3	n=3	n=3
Dab2	35.09 ± 0.88	11.2 ± 1.44	46.29 ± 2.13
n	n=3	n=3	n=3
Cyr61	33.27 ± 1.76	12.87 ± 2.00	46.13 ± 0.48
n	n=3	n=3	n=3
P-Values			
ApoE v's Control	0.6346 (ns)	0.0208 (*)	0.6831 (ns)
Dab2 v's Control	0.0376 (*)	0.07 (ns)	0.6831 (ns)
Cyr61 v's Control	0.1549 (ns)	0.2897 (ns)	0.6491 (ns)

Table S6: Quantification of phenotypic changes upon overexpression of predicted Tead targets, ApoE, Dab2 and Cyr61 (refers to Fig. 7).

a. Table showing the distribution of transfected cells upon overexpression of ApoE, Dab2 and Cyr61, compared to GFP control. **b.** Table showing quantification for $Pax6^+GFP^+$ cells upon overexpression ApoE, Dab2 and Cyr61, compared to GFP control. **c.** Table showing quantification for $Tbr1^+GFP^+$ cells upon overexpression of ApoE, Dab2 and Cyr61, compared to GFP control. **d.** Table showing quantification for $Ctip2^+GFP^+$ cells upon overexpression of ApoE, Dab2 and Cyr61, compared to GFP control. **e.** Table showing quantification for $Satb2^+GFP^+$ cells upon overexpression of ApoE, Dab2 and Cyr61, compared to GFP control.
